# Plant cell wall glycosyltransferases: High-throughput recombinant expression screening and general requirements for these challenging enzymes

**DOI:** 10.1371/journal.pone.0177591

**Published:** 2017-06-09

**Authors:** Ditte Hededam Welner, David Shin, Giovani P. Tomaleri, Andy M. DeGiovanni, Alex Yi-Lin Tsai, Huu M. Tran, Sara Fasmer Hansen, Derek T. Green, Henrik V. Scheller, Paul D. Adams

**Affiliations:** 1 Joint BioEnergy Institute, Emeryville, California, United States of America; 2 Molecular Biophysics and Integrated Bioimaging Division, Lawrence Berkeley National Laboratory, Berkeley, California, United States of America; 3 Environmental Genomics and Systems Biology Division, Lawrence Berkeley National Laboratory, Berkeley, California, United States of America; 4 Biological and Engineering Sciences Center, Sandia National Laboratories, Livermore, California, United States of America; Griffith University, AUSTRALIA

## Abstract

Molecular characterization of plant cell wall glycosyltransferases is a critical step towards understanding the biosynthesis of the complex plant cell wall, and ultimately for efficient engineering of biofuel and agricultural crops. The majority of these enzymes have proven very difficult to obtain in the needed amount and purity for such molecular studies, and recombinant cell wall glycosyltransferase production efforts have largely failed. A daunting number of strategies can be employed to overcome this challenge, including optimization of DNA and protein sequences, choice of expression organism, expression conditions, co-expression partners, purification methods, and optimization of protein solubility and stability. Hence researchers are presented with thousands of potential conditions to test. Ultimately, the subset of conditions that will be sampled depends on practical considerations and prior knowledge of the enzyme(s) being studied. We have developed a rational approach to this process. We devise a pipeline comprising *in silico* selection of targets and construct design, and high-throughput expression screening, target enrichment, and hit identification. We have applied this pipeline to a test set of *Arabidopsis thaliana* cell wall glycosyltransferases known to be challenging to obtain in soluble form, as well as to a library of cell wall glycosyltransferases from other plants including agricultural and biofuel crops. The screening results suggest that recombinant cell wall glycosyltransferases in general have a very low soluble:insoluble ratio in lysates from heterologous expression cultures, and that co-expression of chaperones as well as lysis buffer optimization can increase this ratio. We have applied the identified preferred conditions to Reversibly Glycosylated Polypeptide 1 from *Arabidopsis thaliana*, and processed this enzyme to near-purity in unprecedented milligram amounts. The obtained preparation of Reversibly Glycosylated Polypeptide 1 has the expected arabinopyranose mutase and autoglycosylation activities.

## Introduction

Plant cell walls represent one of the most abundant resources on earth. The ability to control cell wall molecular composition holds great potential in applications from biofuel production to food and feed crop optimization. For example, being able to control the xylan content in biofuel feedstocks would help overcome challenges in recalcitrance and fermentation posed by xylan-rich biomass [1]. The cell wall composition is primarily dictated by biosynthetic Leloir glycosyltransferases [2], the vast majority of which have proven elusive to molecular characterization. These enzymes catalyze the transfer of a sugar moiety from a nucleotide sugar donor to an acceptor, forming a glycosidic linkage. Leloir glycosyltransferases are widespread in nature and play crucial roles in diverse processes in physiology and pathology across the kingdoms of life. Leloir glycosyltransferases involved in plant cell wall polysaccharide and glycoprotein biosynthesis (CWGTs) are numerous. The majority of CWGTs are Golgi-localized or associated, and bioinformatic analyses indicate that most of them are type II membrane-bound glycoproteins [3]. A notable exception is the Reversibly Glycosylated Polypeptides (RGPs) described later.

The described CWGT features could account for the difficulty researchers experience when trying to express the CWGTs recombinantly. First, the transmembrane domain must be deleted, introducing a human-defined N-terminus, which can potentially be problematic for expression levels and solubility. Alternatively, the full-length CWGTs must be treated as membrane proteins, although the majority of their sequence is luminal. This approach is conceivably also very challenging, as demonstrated by the fact that—to the best of our knowledge—no intact type II membrane protein structure has been solved to date. Second, specific chemical and biological components of the plant Golgi lumen, where translation and folding of CWGTs take place in nature, might be necessary for correct folding and solubility. Therefore, researchers might be required to mimic this environment in their heterologous expression system to obtain appreciable amounts of soluble CWGTs. However, to date, such specific factors have not been identified and to the best of our knowledge, have not been the subject of a dedicated study.

It is likely that CWGTs are subject to post-translational modifications in the plant secretory pathway. For example, glycosylation can be key to protein function and solubility [4], and although 6 of the screened enzymes (Table 1, the 3 RGP1 proteins and 3 PARVUS proteins) have no N-glycosylation consensus motif, the majority of CWGTs have at least one. To approximate the glycosylation pattern that native plant CWGTs would have, we have explored secreted recombinant CWGT expression in eukaryotic systems, including the yeast *Pichia pastoris* and *Spodoptera frugiperda* (Sf9) insect cell culture. These hosts were capable of producing high amounts of recombinant CWGT, but the vast majority was found in the insoluble lysis fraction. Such high yields of predominantly insoluble CWGT without significant amounts of soluble target mirrors our experiences with the same sequences in *Escherichia coli* (for examples, see S1 Fig).

**Table 1:** Alphabetical list of the CWGTs that were screened.

Protein*	Gene identifier	CAZy/DUF**	Source organism
ARAD1	At2g35100	GT47	*Arabidopsis thaliana* (thale cress)
ARAD2	At5g44930	GT47	*Arabidopsis thaliana* (thale cress)
EMB2756	100797815	DUF616	*Glycine max* (soy bean)
FUT6	At1g14080	GT37	*Arabidopsis thaliana* (thale cress)
GALS1	At2g33570	GT92	*Arabidopsis thaliana* (thale cress)
GALS1	100787815	GT92	*Glycine max* (soy bean)
Galt31A	At1g32930	GT31	*Arabidopsis thaliana* (thale cress)
GAUT1	At3g61130	GT8	*Arabidopsis thaliana* (thale cress)
GAUT7	At2g38650	GT8	*Arabidopsis thaliana* (thale cress)
GlcAT14A	101210575	GT14	*Cucumis sativus* (cucumber)
GUT1/IRX10L	At5g61840	GT47	*Arabidopsis thaliana* (thale cress)
GUT1/IRX10L	100783737	GT47	*Glycine max* (soy bean)
GUT1/IRX10L	100794632	GT47	*Glycine max* (soy bean)
GUX1	At3g18660	GT8	*Arabidopsis thaliana* (thale cress)
IRX7	At2g28110	GT47	*Arabidopsis thaliana* (thale cress)
IRX8	At5g54690	GT8	*Arabidopsis thaliana* (thale cress)
IRX9	At2g37090	GT43	*Arabidopsis thaliana* (thale cress)
IRX9L	At1g27600	GT43	*Arabidopsis thaliana* (thale cress)
IRX9L	606306	GT43	*Hordeum vulgare* (barley)
IRX14	At4g36890	GT43	*Arabidopsis thaliana* (thale cress)
IRX14	100777505	GT43	*Glycine max* (soy bean)
MGD2	At3g48820	GT29	*Arabidopsis thaliana* (thale cress)
MUCI10	101291758	GT34	*Fragaria vesca* (strawberry)
MUCI10	4350696	GT34	*Oryza sativa* (rice)
MUR3	At2g20370	GT47	*Arabidopsis thaliana* (thale cress)
NN***	At1g53290	GT31	*Arabidopsis thaliana* (thale cress)
DUF246	101299994	DUF246	*Fragaria vesca* (strawberry)
DUF246	101311123	DUF246	*Fragaria vesca* (strawberry)
DUF246	101776477	DUF246	*Setaria italic* (foxtail millet)
PARVUS	At1g19300	GT8	*Arabidopsis thaliana* (thale cress)
PARVUS	101228909	GT8	*Cucumis sativus* (cucumber)
PARVUS	4336486	GT8	*Oryza sativa* (rice)
RGP1	At3g02230	GT75	*Arabidopsis thaliana* (thale cress)
RGP1	100282614	GT75	*Zea mays* (corn)
RGP1	100836426	GT75	*Brachypodion distachyon* (stiff brome)
RGXT2	At4g01750	GT77	*Arabidopsis thaliana* (thale cress)
RRA	100255856	GT77	*Vitis vinifera* (grape)
RRA2	At1g75110	GT77	*Arabidopsis thaliana* (thale cress)
DUF288	101253530	DUF288	*Solanum lycopersicum*
DUF288	101309981	DUF288	*Fragaria vesca* (strawberry)
DUF288	101510562	DUF288	*Cicer arietinum* (chickpea)
TBL13	100802467	DUF231	*Glycine Max* (soy bean)
TBL29	100825801	DUF231	*Brachypodion distachyon* (stiff brome)
XEG113	100819032	GT77	*Glycine max* (soy bean)
XXT1	At3g62720	GT34	*Arabidopsis thaliana* (thale cress)
XXT1	101262652	GT34	*Solanum lycopersicum* (tomato)

46 CWGT sequences comprising 22 *A*. *thaliana* genes and 24 genes from other plants. These sequences were screened for optimal *E*. *coli* expression using the high-throughput screening pipeline established in this study.

* for uncharacterized gene sequences, protein name is inferred from sequence similarity to characterized proteins.

** proteins from four Domain of Unknown Function (DUF) protein families were included in this study since they likely represent hitherto un-recognized CWGTs [5] or cell wall polysaccharide O-acetyltransferases.

*** NN = no name annotated in TAIR (http://www.arabidopsis.org, [6]). Will be referred to as At1g53290 in this text.

Yields of recombinant protein from eukaryotic expression systems are rarely comparable to those obtained with *E*. *coli*, which can grow to very high cell densities [7]. In addition, transformation and culturing methods are generally more demanding and expensive. Since, in our hands, yeast and insect cell expression systems suffer from the same limitations to soluble CWGT production as *E*. *coli*, we set out to systematically explore the full potential for CWGT production in *E*. *coli*, combining several approaches. Firstly, to avoid the pitfall of an individual CWGT being significantly more challenging than the average, and to be able to draw general conclusions about CWGT behavior as recombinant proteins, we screened a large number of genes, including well-characterized, partly characterized, and putative CWGTs from *A*. *thaliana* as well as several crops. Each gene was included in full-length and in several truncated forms, to minimize errors introduced in N-terminal truncation as well as incorrectly predicted gene boundaries and other bioinformatic shortcomings. Secondly, as CWGTs typically contain several cysteines, redox state control could prove key to successful protein folding and thus soluble heterologous expression. Notably, the secretory pathway, including the Golgi lumen, provides a more oxidizing environment than the cytosol [8], where recombinant protein translation and folding typically takes place in *E*. *coli*. To explore this parameter, we included the *E*. *coli* Origami^™^ strain in our screen. This cell line has mutations in the thioredoxin reductase and glutathione reductase genes, enhancing disulfide bond formation in the cytoplasm [9]. We also included a vector for periplasmic expression, since the periplasm is the most oxidizing cellular compartment for protein expression available in *E*. *coli* [10]. In the same vein we included an expression vector that produces a fusion protein with thioredoxin. Thioredoxin has been shown to mediate correct folding of recombinant proteins by proximity, either acting as a chaperone or through its oxidoreductase activity [11]. Finally, the availability and stoichiometry of aids to folding, such as chaperones, will be different in the Golgi lumen compared to the cytosol [12, 13]. To compensate for these differences, we screened co-expression of several combinations of chaperones, including GroEL/GroES, DnaJ/DnaK/GrpE and Trigger Factor.

We have applied this multidimensional screen to a total of 46 CWGTs (Table 1) from 12 different plants covering 11 CAZy families (http://www.cazy.org, [14]) and 4 DUF families (http://pfam.xfam.org, [15]). Gene identity and protein family were not correlated with the observed soluble:insoluble ratio. The general observations were that periplasmic expression and fusion with thioredoxin did not increase CWGT solubility. This indicates that redox control alone cannot alleviate the limitations in soluble recombinant CWGT production. In contrast, co-expression with chaperones, including DnaK/DnaJ/GrpE and Trigger Factor, seemed to enhance soluble CWGT production. We therefore conclude that correct folding of CWGTs in heterologous expression hosts is problematic and that this bottleneck can be partly alleviated by co-expression with chaperones.

RGP1 is the only cytosolic protein included in the screen. *A*. *thaliana* RGP1 has previously been heterologously expressed as a glutathione S-transferase fusion protein [16] and alone in *E*. *coli*(DE3) Star^™^ [17] but with very limited yield. RGPs have been found in association with the cytosolic side of the Golgi membrane [18] and do not have a transmembrane domain, which might account for the relative success of recombinant RGP1 expression. This class of proteins is involved in cell wall biosynthesis through arabinopyranose mutase activity, interconverting the pyranose and furanose forms of arabinose, arabinofuranose being incorporated into the plant cell wall. In addition, RGPs are reversibly autoglycosylating, an activity that is believed to be secondary to the mutase activity and may play a regulatory role [16–19].

## Results

With the ambition to build a knowledgebase of CWGT molecular properties, two clone libraries were constructed. These were fed into a high-throughput screening pipeline for recombinant CWGT expression, using a fractional experimental design to allow testing of a large–but not exhaustive–number of conditions. First, a test library of in-house *Arabidopsis thaliana* CWGT clones were employed to design and implement the pipeline. Then, a second library of non-*Arabidopsis* CWGTs was fed into the pipeline, with the experimental parameters, including the vector panel, optimized based on the output from the test library screening. This second library was designed based on *in silico* analyses of sequence similarities, cysteine content, and structure, disorder, and glycosylation site predictions, with the goal to identify promising CWGT candidates from important crops and biofuel feedstocks for molecular characterization.

### Test CWGT library and screening pipeline set-up

The test library was constructed of in-house clones. The aim was to provide a relatively well-characterized set of CWGT sequences for screening pipeline design and implementation. The library includes 22 CWGT genes from the model plant *Arabidopsis thaliana* (Table 1). In addition to the full-length genes, truncated sequences were included, omitting the N-terminal transmembrane domain where relevant. In addition to the N-terminal truncations, we removed predicted [20] unstructured C-terminal regions from 3 of the constructs yielding a total of 38 clones (S1 Table).

We designed a high-throughput cloning strategy using Gateway^™^ cloning into *E*. *coli* expression vectors that provide a histidine tag for purification. We combined this with small-scale transformation in PCR plates and expression in 1 mL cultures in 96 deep-well blocks. For cell lysis, we tested plate sonication but this resulted in inhomogeneous lysis through the plate. Instead, we opted for detergent-mediated lysis. To ensure that the detergent would not give false positives resulting from detergent-mediated solubilization of otherwise insoluble target material, we did a side-by-side comparison with detergent and sonication of a sample of randomly selected targets. This analysis demonstrated no solubilizing effect of detergent. In fact, sonication yielded slightly more soluble target (S2 Fig).

**Fig 1:**
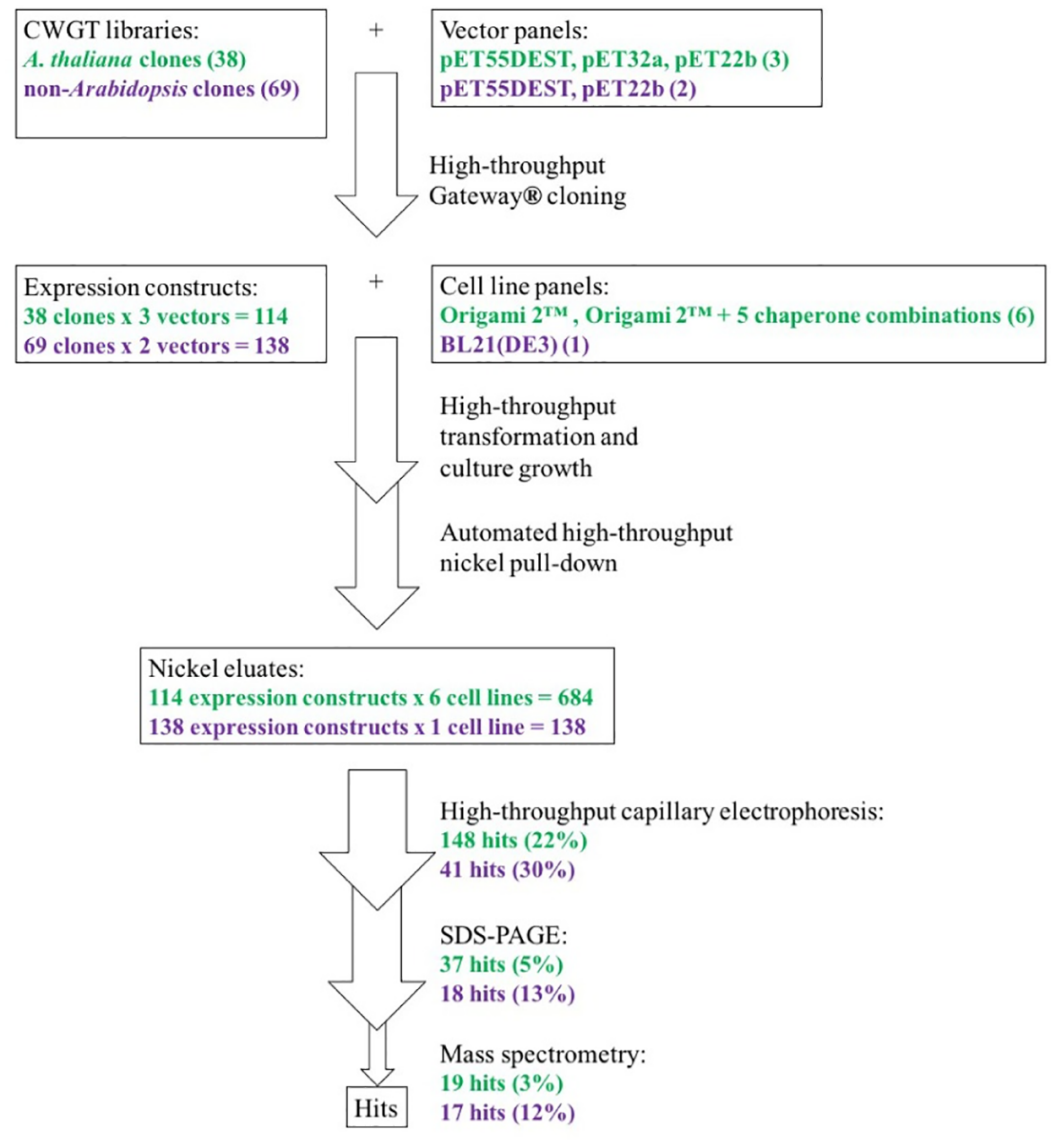
Flow diagram of the high-throughput expression screening pipeline.

*A priori*, we expected a very low soluble:insoluble ratio, with no detectable band above background on a Coomassie-stained SDS-PAGE of the soluble lysate fraction. We opted to avoid highly sensitive analysis of the soluble lysate fraction such as activity detection or western blotting, since we have in the past found the minute amounts of soluble target that these methods can detect not to be scalable to the amounts needed for molecular characterization. Instead, we implemented a high-throughput nickel pull-down protocol using a liquid handling robot, thereby obtaining a concentrated eluate enriched in CWGT. This way we selected for soluble CWGT that can bind to and elute from nickel resin. In addition to being pre-requisite for successful scale-up, this indicates that the CWGT is folded and stable. The eluates were analyzed with automated capillary electrophoresis (Labchip GXII). Samples containing peaks corresponding to protein species of the expected molecular weight, ±15%, were identified in the eluates using the Labchip GXII software, and subjected to SDS-PAGE and mass spectrometry of the gel bands. The general flow of the screening pipeline is given in Fig 1.

We combined the test library (38 clones) with three vectors for intracellular expression, periplasmic expression, and intracellular expression as a thioredoxin fusion protein, respectively. These constructs were transformed into *E*. *coli* Origami 2^™^, as well as *E*. *coli* Origami 2^™^ co-expressing one of 5 different chaperone-encoding plasmids. Out of the resulting 684 samples, the automated capillary electrophoresis software identified bands corresponding to the expected protein weight in 148 (~22%) samples (S3 Table). 37 samples (~5% of total) gave one or more appreciable band(s) above background on SDS-PAGE (S2 Data). Based on this rather limited success rate (37 of 148), we decided to employ concentration and purity cutoffs in the next library screening (see next section). The identity of the SDS-PAGE band was confirmed by mass spectrometry for 19 (~2.8%) samples (Table 2, S1–S4 Data). At1g53290 and RGP1 each represent 4 of the 19 hits, IRX9 represents 3, GUT1/IRX10L and XXT1 represent 2 each, and a total of 10 (out of 22) unique CWGTs are represented in the final set of hits (Table 2). Of the 19 hits, 11 are full-length and 8 are N-terminally truncated, suggesting that truncation of the transmembrane domain increases solubility for some CWGTs, but is not a general requirement. However, a closer look at the 11 full-length sequences reveals that 4 of them are RGP1 –which does not have a transmembrane domain–and 6 are thioredoxin fusion proteins, which turned out to precipitate upon cleavage as described below and hence probably do not represent truly folded, soluble CWGTs. The only full-length hit with a transmembrane domain and without thioredoxin is At1g53290.

**Table 2:** Hits from the test library screening.

Protein	Construct	Vector	Chaperone co-expression	Yield (μg/mL)
At1g53290	Δ1–50	pET55dest	DnaK, DnaJ, GrpE	5.3
At1g53290	full-length	pET55dest	DnaK, DnaJ, GrpE	18.0
At1g53290	Δ1–50	pET32dest	DnaK, DnaJ, GrpE	1.2
At1g53290	full-length	pET32dest	Trigger Factor	0.4
FUT6	Δ1–12 and Δ507–519	pET22dest	Trigger Factor	1.8
Galt31A	full-length	pET32dest	Trigger Factor	0.6
GUT1/IRX10L	Δ1–46	pET55dest	DnaK, DnaJ, GrpE	0.6
GUT1/IRX10L	Δ1–46	pET32dest	Trigger Factor	1.0
IRX9	full-length	pET32dest	DnaK, DnaJ, GrpE	4.6
IRX9	full-length	pET32dest	Trigger Factor	0.8
IRX9	Δ1–72	pET55dest	Trigger Factor	7.0
MUR3	Δ1–100	pET55dest	DnaK, DnaJ, GrpE	6.8
PARVUS	Δ1–39	pET55dest	Trigger Factor	1.6
RGP1	full-length	pET55dest	none	111.0
RGP1	full-length	pET55dest	GroEL, GroES	271.8
RGP1	full-length	pET32dest	Trigger Factor	1.3
RGP1	full-length	pET32dest	DnaK, DnaJ, GrpE	1.6
XXT1	full-length	pET32dest	Trigger Factor	9.6
XXT1	full-length	pET32dest	DnaK, DnaJ, GrpE	10.9

Periplasmic targeting is not beneficial to this set of CWGTs under the given expression conditions, giving only 1 hit with a limited yield (1.8 μg/mL of FUT6). pET55dest and pET32dest produce comparable numbers of hits (8 and 10) with a notably higher average yield for the pET55dest clones (52 μg/mL, and 3 μg/mL, respectively). As mentioned, experiments later showed that the pET32dest thioredoxin fusion proteins precipitated upon cleavage, leaving only a very small fraction of the cleaved CWGT soluble (see S3 Fig for an example). Consequently, we deemed the pET32DEST vector irrelevant for further screening. Finally, this first screening indicates that co-expression of certain chaperones can be helpful, with Trigger Factor and a combination of DnaK, DnaJ and GrpE showing similar hit rates (9 and 8, respectively) and accounting for 17 of the 19 hits, each giving yields in the 3 μg/mL range. However, this is not comparable to the 1 hit without chaperone co-expression, RGP1 in pET55dest (111 μg/mL), or the yield of RGP1 with GroEL/GroES co-expression (271.8 μg/mL), indicating that yields of targets requiring chaperone co-expression might turn out to be insufficient for molecular characterization such as protein structure determination.

**Fig 2:**
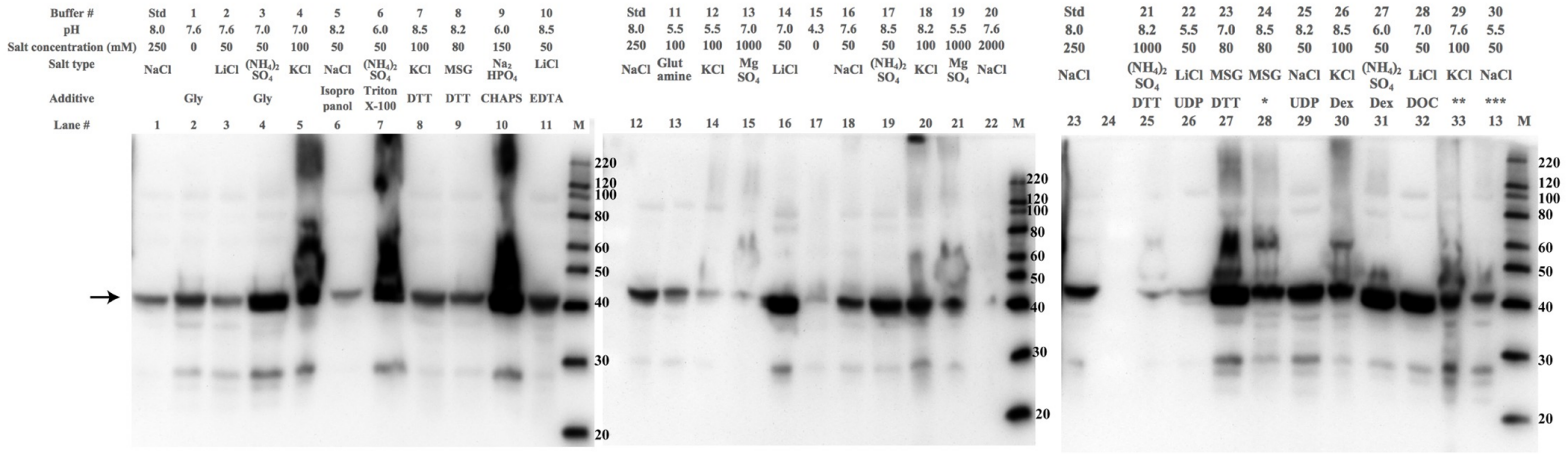
The effect of salts, pH and additives in the lysis buffer on the solubility of RGP1. Western blots against a cloning scar shows the varying amount of RGP1 in the soluble lysis fraction after lysing in different buffers. The arrow indicates the band corresponding to full-length RGP1 (42 kDa). Buffers 13 and 19–21, containing 1-2M salt, as well as the low-pH (4.3–5.5) buffers 11,12,15, 22 and 30 decrease the amount of soluble RGP1, while lower salt concentrations as well as several additives increase the amount of soluble RGP1 relative to the standard lysis buffer (std: 50 mM HEPES pH 8.0, 250 mM NaCl, 5 mM MgCl_2_). MSG = monosodium glutamate, Dex = dextran. *Condition 24: 100 mM triethanolamine, 80 mM sodium glutamate, 0.02% n-octyl-β-D-glucoside, 10% glycerol, 5mM MgSO_4_, pH 8.5. **Condition 29: 100 mM Tris, 100 mM KCl, 0.1% deoxycholate, 25% glycerol, pH 7.6. ***Condition 30: 100 mM potassium acetate, 50 mM NaCl, 0.05% dextran sulfate, 0.1% CHAPS, pH 5.5.

The described pipeline enables identification of promising CWGTs, optimal expression vector and beneficial chaperones for co-expression. These strategies can enhance the recovery of soluble CWGT. However, the majority of the recombinant material is still insoluble for all the CWGTs we have investigated. In an effort to further shift the equilibrium towards the soluble fraction, we employed a lysis buffer screen based on the work of Lindwall and colleagues [21]. The screen includes 30 lysis buffers containing salts of varying chaotropy, different pH, and additives. We added magnesium sulfate to a majority of the buffers, since the magnesium ion is a co-factor for many nucleotide sugar-binding enzymes. We also added uridine diphosphate (UDP, a CWGT product) to 10% of the buffers. The list of customized buffers is given in S1 List. We then tested the lysis buffer screen on RGP1. We found that lysis buffer composition had a significant impact on the amount of RGP1 obtained in the soluble lysis fraction, although this effect could not be accurately quantified by the visual inspection of western blots that we employed as readout. In particular, low pH and high salt had a strong negative effect on solubility. Absence of salt, and moderate salt concentrations (50–150 mM), were beneficial, while the salt type did not have a discernible effect. Among the additives, several seemed to have a neutral-to-positive effect. The described effects are illustrated in Fig 2. In conclusion, we found the optimal lysis buffer for RGP1 to be 100 mM HEPES pH 7.0, 50 mM LiCl_2_, 0.1% deoxycholate.

### *In silico* selection and high-throughput expression screening of non-*Arabidopsis* CWGTs

Studies of CWGTs from *Arabidopsis thaliana* are over-represented in the literature compared to other plants, due to this organism’s pliability as a model plant and the availability of extensive sequencing data. Unfortunately, it is not an agricultural or biofuel crop. This, in combination with the well-established difficulty of obtaining the *Arabidopsis thaliana* CWGTs in sufficient amounts for molecular characterization, prompted us to look to crop and biofuel feedstocks for other CWGTs. We decided to employ a bioinformatics strategy to select sequences that theoretically would have the highest probability of producing soluble protein in an *E*. *coli* expression system.

A shortlist of 43 CWGT sequences from *Arabidopsis thaliana* (S2 Table) was generated, based on published and in-house experimental evidence of their CWGT function and so aimed to efficiently exclude any non-translated DNA sequences and pseudo proteins. This list formed the basis for DELTA-BLAST [22] searches. From these searches, 10750 targets were found among >100 different species. The top 3 non-*Arabidopsis* species with the most targets were *Zea mays*, *Glycine max* and *Oryza sativa*. We further culled our target sequences to select those that had a minimum of 40% sequence identity to the parent query sequences. We choose this low cutoff to get diversity. We then filtered out target sequences shorter than 85% the length of the parent sequence, to likely select full-length sequences, as well as target sequences longer than 115% the parent sequence length, to reduce the number of targets with extra domains, which may be evolutionarily distinct and possess different functions than the parent sequence. To minimize the effects of altered or missing glycosylation and aberrant disulfide bridges on heterologous expression and solubility, we ordered the protein sequences according to the number of putative N-glycosylation sites (0–12) and cysteines (1–18). Target sequences were then binned according to how their query sequences sorted in a phylogenetic tree (S4 Fig). Ten bins were selected by visually inspecting the tree, and choosing groups by their evolutionary distances. Finally, to aid in designing clones for expression screening, each protein sequence was annotated with information about 1) predicted N-terminal transmembrane domains, 2) potential disordered domains, and 3) predicted secondary structure elements (S1 Pred).

**Fig 3:**
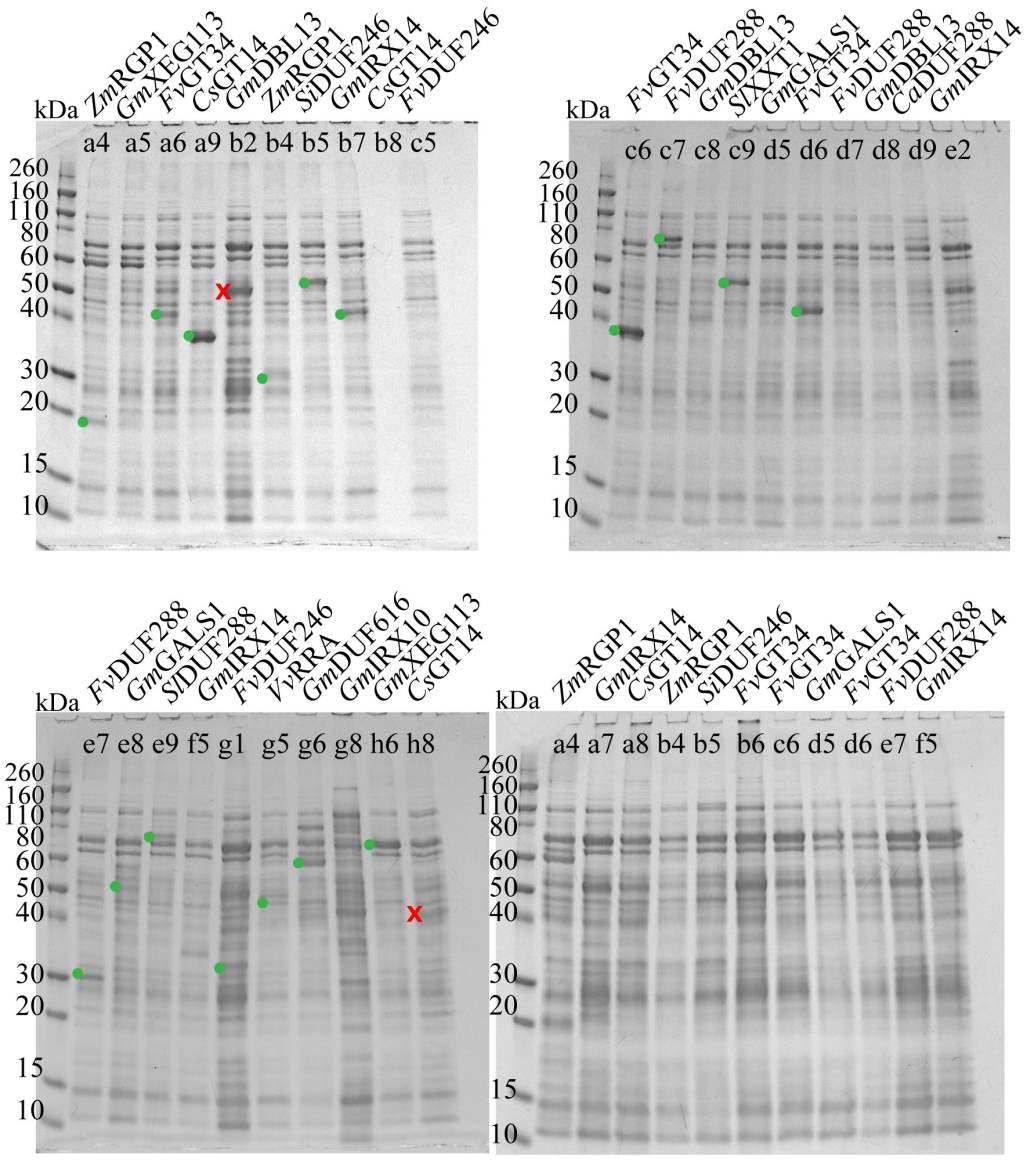
SDS-PAGE of selected samples from the non-*Arabidopsis* library. Coomassie-stained gels showing nickel affinity eluates of samples selected from the automated capillary electrophoresis procedure as described. Each lane is marked with the organism and protein name, as well as with the well number for reference to S6 Data. Bands that were excised for mass spectrometry are marked with a green dot (confirmed by mass spectrometry) or a red cross (not confirmed). The lower right gel has samples expressed in the periplasm (pET22DEST vector, no excisions), while the other three gels have samples expressed in the cytoplasm (pET55DEST vector). Sample b8 (upper left gel) has no detectable background on SDS-PAGE, but produces a normal band pattern in the automated capillary electrophoresis, indicating that the absence of bands on SDS-PAGE is likely due to faulty well loading. *Ca = Cicer arietinum*, *Cs = Cucumis sativa*, *Fv = Fragaria vesca*, *Gm = Glycine max*, *Si = Setaria italica*, *Sl = Solanum lycopersicum*, *Vv = Vitis vinifera*, *Zm = Zea mays*.

A subset of sequences resulting from the *in silico* analysis was input to the expression screening pipeline for proof-of-concept. These sequences were chosen from the above described target sequences to cover as many non-*Arabidopsis* plants as possible and all 10 of the phylogenetic groups. Within these restrictions, sequences with the lowest possible numbers of cysteines and putative N-glycosylation sites, and with expression evidence (EST, mRNA, cDNA) were selected for the non-*Arabidopsis* library. This selection procedure resulted in 24 unique CWGTs from 11 different plants. These were included in full-length and in truncated versions, as suggested by the *in silico* analyses described above (S1 Pred). A total of 69 constructs (S4 Table) were expressed intracellularly or in the periplasm of *E*. *coli* BL21(DE3). The pET32dest vector was omitted because it produced false positives in the test screen. Further, chaperone co-expression was omitted, to simplify the output and because the test screen indicated that while some chaperones did indeed solubilize some CWGTs, the yield was not in the range needed for molecular characterization. Because of the relatively high number of false positives resulting from the automated analysis of the test library screen (only 19 samples were confirmed by mass spectrometry, but 148 samples were analyzed with SDS-PAGE, see above) we decided to employ a cutoff on the concentration and purity parameters in the Labchip GXII analysis. 25 samples yielding a peak corresponding to the expected protein weight and with a purity above 27% or a concentration above 70 μg/mL were selected for SDS-PAGE (tab ‘cutoffs’ in S6 Data). To minimize the risk of false negatives, we supplemented this automated approach with visual inspection and selection in the Labchip GXII ‘gel mode’ (tab ‘Labchip GHII results—gels’ in S6 Data), selecting additionally 16 samples (S5 Table). SDS-PAGE and mass spectrometry confirmed the identity of 17 bands (~12% of the screened samples) (Fig 3, Table 3 and S5 Data). These 17 samples in general gave higher yields than those obtained from the test library (averages of 68 and 28 μg/mL, respectively) indicating an optimization of expression conditions. Of the 11 species represented in the library, 7 species gave at least one hit and there is no apparent correlation between species and success rate. Out of the 17 hits, RGP1 is represented in two truncated forms of an expressed sequence tag from *Zea mays* (locus 100282614) with 89% identity to *Arabidopsis thaliana* RGP1. None of the 17 hits are full-length, indicating that truncation increases the solubility for most CWGTs. This is in line with our observation from the test library that 6 of the 7 full-length hits (not counting the 4 hits from the non-transmembrane RGP1 protein) were false positives. Finally, corroborating the trend seen with the test library, periplasmic expression did not yield any hits. Based on the SDS-PAGE analysis shown in Fig 3 the most promising targets in terms of amount and purity in the nickel eluate are N- and C-terminally truncated versions of a GT14 from *Cucumis sativus* (well a9, gene locus 101210575, residues 30–342) and GT34 from *Fragaria vesca* (well c6, gene locus 101291758, residues 92–392).

**Table 3:** Hits from the non-*Arabidopsis* library screening.

ID	Protein	Construct	Species	Yield (μg/mL)
D02_Clone01	ZmRGP1 (a4)	Δ135–361	*Zea mays*	30.3
D04_Clone01	FvMUCI10 (a6)	Δ1–91	*Fragaria vesca*	35.9
D18_Clone03	CsGlcAT14A (a9)	Δ1–29 and Δ343–396	*Cucumis sativus*	86.3
D02_Clone02	ZmRGP1 (b4)	Δ237–361	*Zea mays*	23.5
D08_Clone01	SiDUF246 (b5)	Δ1–63	*Setaria italic*	24.3
D13_Clone03	GmIRX14 (b7)	Δ1–78 and Δ436–502	*Glycine max*	119.8
D04_Clone03	FvMUCI10 (c6)	Δ1–91 and Δ393–457	*Fragaria vesca*	120.6
D22_Clone01	FvDUF288 (c7)	Δ1–95	*Fragaria vesca*	110.8
D03_Clone01	SlXXT1 (c9)	Δ1–46	*Solanum lycopersicum*	40.2
D04_Clone04	FvMUCI10 (d6)	Δ1–57 and Δ393–457	*Fragaria vesca*	223.5
D22_Clone03	FvDUF288 (e7)	Δ1–95 and Δ311–761	*Fragaria vesca*	45.9
D06_Clone02	GmGALS1 (e8)	Δ1–49	*Glycine max*	21.3
D21_Clone01	SlDUF288 (e9)	Δ1–104	*Solanum lycopersicum*	56.2
D13_Clone04	GmIRX14 (f5)	Δ1–156 and Δ436–502	*Glycine max*	94.8
D23_Clone01	VvRRA (g5)	Δ1–53	*Vitis vinifera*	63.3
D20_Clone01	GmEMB2756 (g6)	Δ1–104	*Glycine max*	20.0
D24_Clone01	GmXEG113 (h6)	Δ1–52	*Glycine max*	41.2

The hits are ordered like in Fig 3 and marked with the well number for easy referral. The ID refers to S4 Table.

### Expression scale-up and protein purification

**Fig 4:**
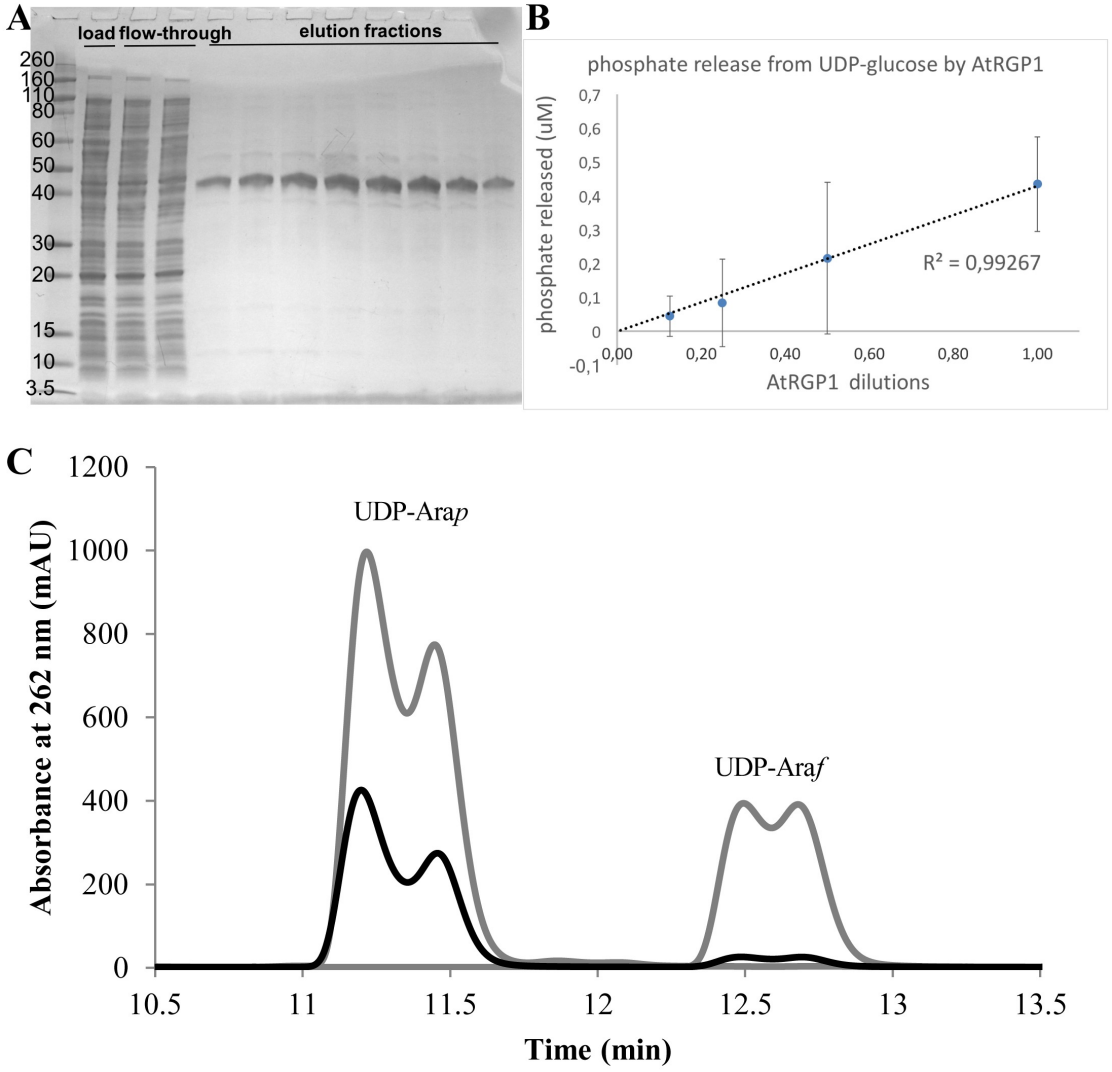
*Arabidopsis thaliana* RGP1 scale-up, purification and activity determinations. (A) Coomassie stained SDS-PAGE of the final chromatographic step yielding almost pure RGP1 (42 kDa). (B) Phosphate-release assay showing autoglycosylating or hydrolytic activity of RGP1 on UDP-glucose. (C) UDP-arabinose mutase activity of RGP1. High-pressure liquid chromatograms of authentic UDP-arabinopyranose (UDP-Ara*p*) and UDP-arabinofuranose (UDP-Ara*f*) standards (grey) overlaid with the chromatogram of the reaction mixture of UDP-Ara*f* with recombinant, purified RGP1 (black).

To investigate scalability and validate our approach, we expressed *Arabidopsis thaliana* RGP1 in 2 L of *E*. *coli* Origami^™^ culture and purified it to near-homogeneity (Fig 4A) with a final yield of ~1 mg of purified RGP1. This is an advancement compared to previous purification attempts, which gave very low yields that required a highly sensitive fluorescent dye for detection [17] or expression as a fusion protein [16]. We demonstrated that the preparation has the expected mutase (Fig 4B) [17] and autoglycosylation (Fig 4C) [16] activities.

## Discussion

We have designed a high-throughput pipeline for *E*. *coli* expression screening of recombinant proteins that have proven difficult to obtain in significant amounts from standard *E*. *coli* expression conditions. The pipeline includes *in silico* target selection and construct design, a multidimensional screen enabling panels of vectors and expression strains to be tested, and high-throughput nickel pull-down and automated capillary electrophoresis for accurate and fast readout. The pipeline employs a fractional experimental design to allow screening most of the factors known to influence recombinant protein expression. We opted for this, since a full factorial design would have generated a prohibitively large number of samples, or severely restricted the number of parameter feasible to test. We have employed this pipeline to investigate recombinant expression of plant CWGTs. The pipeline is easily adaptable to other types of protein targets presenting other challenges, and so will be a versatile tool for experiments that require milligram quantities of isolated protein.

The CWGTs investigated in this study benefitted from the co-expression of Trigger Factor and DnaK, DnaJ, GrpE chaperones, indicating that correct CWGT folding in the *E*. *coli* cytosol represents a bottleneck in recombinant CWGT production. Expression as thioredoxin fusion proteins, as well as periplasmic expression did not enhance soluble CWGT expression, which could indicate that redox control is not the limiting factor in recombinant CWGT folding.

Interestingly, the cytosolic and Golgi-associated RGP1, which constituted 5% of the total number of constructs tested, produced 6 out of 37 hits identified from the two libraries (~16%). This can be interpreted in multiple ways. First, it could indicate that features intrinsic to individual CWGTs are decisive, from which it follows that it cannot be assumed that all members of this class of enzymes pose the same challenges. RGP1 could simply be easier to express than “the average CWGT”. This hypothesis is supported by the fact that other CWGTs are also over-represented amongst the hits; At1g53290 and homologues from other species constituted ~2% of the total constructs and ~11% of the hits, and XXT1 constituted ~4% of the total constructs, and ~8% of the hits. Second, the relative success of RGP1 recombinant expression could be attributed to its native environment–the plant cytosol–conceivably being more similar to the *E*. *coli* cytosol than the plant Golgi lumen where the other tested CWGTs reside. The molecular mechanism underlying this can only be speculated at, but the present data indicate that redox control cannot explain it alone. Finally, the absence of a transmembrane domain in RGP1 could be a major contributing factor. This CWGT is expressed as full-length, and thus its N-terminus was not manipulated, but rather defined by nature. If this is in fact the fundamental problem for the other CWGTs, which all have an N-terminal transmembrane domain, effort should be placed in the design of N-terminal truncations and screening different truncation sites in the future.

While we have succeeded in overexpressing and isolating individual CWGTs using the pipeline described here, the majority of the expressed recombinant material is still present in the insoluble lysis fraction. Hence, while we are able to tune the soluble:insoluble fraction somewhat, we have not identified a “silver bullet”. There are a couple of remaining factors to explore: On the supramolecular level, it is possible that an individual CWGT is not soluble or cannot fold properly without a binding partner, since several CWGTs have been shown to exist and function in complexes [3]. This could be investigated by co-expression of, for example, GAUT1/GAUT7 or ARAD1/ARAD2. RGPs from several plants have also been shown to exist and function in complexes [17, 22–25]. Secondly, it is possible that even with chaperone co-expression and redox control, the CWGTs are not correctly folded by the intrinsic *E*. *coli* machinery, and/or that they lack essential eukaryotic post-translational modifications such as glycosylations, which can play a major role in protein solubility [4]. However, we observed very limited success with secreted expression in yeast or insect cells, which would allow for eukaryotic glycosylation and other post-translational modifications. It is possible that yeast and insect cell glycosylation patterns are still too dissimilar to the native glycosylation pattern. Instead, mammalian cell culture could potentially perform better, or even overexpression in a plant or plant tissue culture expression system. In this context, recent advances in algae expression systems could be interesting to explore [26]. Finally, the DNA sequence rather than the protein sequence could play a role in successful soluble expression. While codon optimization to eliminate rare codons is common and we have tested this for CWGT expression, a more nuanced view on codon usage might be beneficial. In the case of CWGTs, expression levels are high, indicating no premature translation arrest or dissociation of the translational machinery due to rare codons. But the dominance of insoluble CWGT production could indicate aberrant folding, and this could be a result of a translational rate beyond the desired. If *in planta*, the CWGT DNA sequences include rare codons at specific sites, allowing ribosomal pausing and hence co-translational folding of domains or isolated secondary structures before translational elongation [27], this built-in regulatory control is likely not replicated in a heterologous host. This could be investigated by a codon harmonization approach [28], where the relative codon frequency of each codon in the source plant is matched in the expression organism. RGP1 does indeed contain 4 codons (corresponding to amino acids 35, 47, 176 and 319) that are more than 5 times as frequent in *E*. *coli* compared to *Arabidopsis thaliana*, which could result in local accelerated translation with resulting aberrant folding.

In conclusion, we have devised a pipeline for high-throughput expression screening of difficult protein targets and applied it to CWGTs. We have found that cytosolic expression with chaperone co-expression can improve recombinant production of CWGTs for molecular characterization. RGP proteins seem to be more amenable to this than other CWGTs tested here, and we have successfully produced ~1 mg of >90% pure *Arabidopsis thaliana* RGP1 that has the expected mutase and autoglycosylation activities.

## Materials and methods

### High-throughput cloning, expression, and nickel pull-down

CWGT sequences were obtained from the TAIR9 genome release (http://www.arabidopsis.org, [6]) and cloned into the spectinomycin-resistant Gateway^™^ donor vector pDONR223 (Thermo Fisher Scientific, cat# 12536017) or purchased in this vector from SGI-DNA (San Diego, USA). In a round-bottomed 96-well plate, 1 μl of each donor vector was mixed with 1 μl of expression vector and 0.25 μl LR Clonase II (Thermo Fisher Scientific, cat# 11791100) and incubated 2 h at room temperature. The tested expression vectors were all from Merck Millipore: pET55DEST (cat# 71846) for intracellular expression, and in-house Gateway-enabled versions of pET32a (cat# 69015) for intracellular expression of thioredoxin fusion protein and pET22b (cat# 69744) for periplasmic targeting. Transformation was achieved by heat shock, adding 2.5 μl of clonase reaction to 25 μl of heat-competent TOP10 cloning cells (Thermo Fisher Scientific, cat# C404010). After a 4 h recovery phase at 37°C in 100 μl SOC medium without antibiotics, the total volume was used to inoculate 1 mL LB medium containing 100 μg/mL carbenicillin in 96 deep-well blocks. The blocks were shaken overnight at 37°C, 900 rpm, and then the cultures were miniprepped using the QIAprep Turbo 96 Miniprep Kit (Qiagen, cat# 27191) and the QIAvac 96 vacuum manifold (Qiagen, cat# 19504). The expression constructs were then transformed into heat-competent expression cells with the above described procedure. The tested expression cell lines were BL21(DE3), and Origami2(DE3)^™^ (Merch Millipore, cat#71408). Antibiotics for Origami2^™^ selection were tetracycline at 10 μg/mL and streptomycin at 50 μg/mL. Expression cell lines pre-transformed with chaperone-encoding plasmids from Chaperone Plasmid Set (Takara, cat#3340) were also tested, and selected for with 20 μg/mL chloramphenicol. 10 μl of the overnight cultures were used to inoculate 1 mL LB-autoinduction medium in 96 deep-well blocks, containing the appropriate antibiotics plus 0.5 mg/mL arabinose for chaperone induction, when relevant. The cultures were shaken at 37°C, 900 rpm until dense, then shifted to 15°C and grown for 24 h. Due to the large number of samples, the OD_600_ was not measured, but cell density was merely estimated by visual inspection of the 1 mL cultures. After harvest, the cells were lysed in 25 mM HEPES pH 8.0, 250 mM NaCl, 5% glycerol, 5 mM MgCl_2_, 50 mM arginine, 50 mM glycine, 1% Triton-X100, 3 μg/mL DNAse I, 100 μg/mL lysozyme, 1 mM PMSF, by shaking at room temperature for 0.5 h. The lysates were spun down at 4000 x *g* for 10 min, and then loaded onto nickel resin in a 96 pipette tip format (20 μl Phytips, PhyNexus, cat# PTB 92-20-03) by pipetting for 0.5 h using a Biomek FXp liquid handling robot. Using the robot, the resin was washed with 360 μl (18 CV) of 25 mM HEPES pH 8.0, 250 mM NaCl, 5% glycerol, 5 mM MgCl_2_, 50 mM arginine, 50 mM glycine, 50 mM imidazole, and the samples were eluted in 50 μl of the same buffer supplemented with 250 mM imidazole.

### Target protein detection by automated capillary electrophoresis and SDS-PAGE

Automated high-throughput capillary electrophoresis was carried out in round-bottomed 96-well plates on a Labchip GXII instrument, using the Protein Express Assay kit and the high-sensitivity method according to the manufacturer’s instructions (PerkinElmer cat# CLS960008). Selected samples were run on SDS-PAGE, using 4–20% Mini-PROTEAN TGX precast gels, which were subsequently stained with InstantBlue (Expedeon, cat# ISB1L, Expedeon).

### RGP1 lysis buffer screen

*E*. *coli* Origami 2^™^ cells transformed with the RGP1-pET55dest plasmid were grown at 37°C in autoinduction medium for 5 h. Then the culture was cooled to 18°C for protein expression and grown for 24 h. Cells were harvested by centrifugation at 5000 x *g* for 10 min at 4°C and the cell pellet was frozen and stored at −80°C. The cell pellet was thawed and resuspended in 30 mL 10 mM Tris pH 8.5, 100 mM NaCl, 1 mM EDTA at 4°C. The resuspension was divided into 31 1 mL portions in 1.5 mL microcentrifuge tubes and again pelleted by centrifugation. This step serves to wash the cells and to apportion them for treatment with the different lysis buffers, and is crucial for reproducibility [21]. Then, the pellets were resuspended in 1 mL of each lysis buffer. 0.15 mg/mL lysozyme and 0.12 mg/mL protease inhibitor (4-(2-Aminoethyl) benzenesulfonyl fluoride hydrochloride (AEBSF)) were added to each sample and the resuspensions were incubated on ice for 5 min. After that, the cells were disrupted by sonication, and the lysate was centrifuged for 10 min. at 16,000 x *g* at 4°C. Equal volumes of each supernatant were analyzed on SDS-PAGE and the level of protein recovery in each condition was assessed by visually comparing the intensity of the bands on a western blot against a cloning scar (see next section).

### Western blot

Electrophoresed samples were blotted onto a nitrocellulose membrane with an iBlot Dry Blotting system (Thermo Fisher Scientific, cat# IB21001). The membrane was blocked for 1 hour in 2% milk powder in phosphate buffered saline. It was then incubated with a 1:10,000 dilution of a custom-made polyclonal rabbit antibody against a C-terminal cloning scar (α-attB2) [29] present in all constructs, in phosphate-buffered saline with 1% fetal bovine serum overnight at 4°C. This was followed by a 1 h room temperature incubation in goat anti-rabbit IgG conjugated with peroxidase (Pierce, #31460) diluted 1:20,000 in phosphate buffered saline with 0.1% TWEEN 20. The blot was developed with SuperSignal West Pico Chemiluminescent Substrate (Thermo Scientific, cat#3 4087) and imaged with a MultiImage III imaging system (Alpha Innotech).

### Bioinformatics

DELTA-BLAST [22] searches were run from an in-house written csh shell script pointing to a local copy of deltablast 2.2.30+ and a non-redundant National Center for Biotechnology Information protein database that was filtered to represent only plant species. The searches were performed on a 2.66 GHz Intel Core i7 Apple MacBook pro with 8 GB of RAM using all 4 cores. The phylogenetic tree was generated by Clustal Omega [30]. Predictions of transmembrane domains were done with Phobius [31] and TM-pred [32]. Disorder prediction were done with GlobPlot [33] and IUPred [34]. Secondary structure elements were predicted with NPS [20], DSC [35], HNN [36], PHD [37], Predator [38], and SIMPA96 [39].

### Phosphate release assay

The phosphate release assay was performed according to [40]. Briefly, purified RGP1 was incubated with 1 mM UDP-glucose (Sigma Aldrich, cat#U4625) and 5 U/mL calf intestinal alkaline phosphatase (New England Biolabs, cat#M290L) at room temperature for 5 h. Phosphates on UDP, exposed by cleavage of the UDP-glucose glycosidic linkage by RGP1, are cleaved off by the phosphatase to form inorganic phosphate, which in turn can be quantified by malachite green, which forms a green complex with inorganic phosphate. The complex was detected at Abs_620 nm_ with a SpectraMax M2 plate reader spectrophotometer. Malachite reagents A and B were prepared as described by Van Veldhoven and Mannaerts [41]. The assay was performed in 96-well clear flat bottomed plates in a total reaction volume of 150 μL consisting of assay buffer (50 mM HEPES pH 7.5, 250 mM NaCl, 5 mM MgCl_2_), calf intestinal alkaline phosphatase, 1 mg/mL henn egg white lysozyme, and RGP1 in serial 2-fold dilutions from 100 μg/mL to 6.25 μg/mL. A standard curve of UDP dilutions (2.5 uM– 0.04 uM) was included in all plates. All reactions were set up in duplicates.

### RGP1 mutase activity assay

UDP-Ara*p* and UDP-Ara*f* were obtained from CarboSource Services (Athens, USA) and Peptide Research Institute (Osaka, Japan), respectively. The RGP1 mutase activity assay and product detection were modified from methods previously described [17]. Briefly, 3 μg recombinant *Arabidopsis thaliana* RGP1 and 1 mM UDP-Ara*f* were added to 20 mM Tris, 5 mM MnCl_2_, 250 mM NaCl, pH 6.8, and incubated at 30°C for 1 h. The product was detected with high-performance anion-exchange chromatography using a Dionex Ultimate 3000 with CarboPac PA20 column (3 × 150 mm; Dionex; Sunnyvale, USA). Nucleotide sugars were separated with 0 to 2.1 min, 50 mM ammonium formate, isocratic; 2.1 to 40 min, 50 mM to 1 M ammonium formate, linear, 40 to 45 min, 1 M to 50 mM ammonium formate, linear, 45 to 55 min, 50 mM ammonium formate, isocratic, all at a flow rate of 0.5 mL/min. Nucleotide sugars were detected with UV light at 262 nm.

## Supporting information

S1 Fig Recombinant CWGTs are mainly found in the insoluble lysis fraction. Coomassie-stained SDS-PAGE of soluble (S) and insoluble (I) lysis fractions from *E*. *coli* expression of 4 different CWGTs. M = marker. CsGT14 = *Cucumis sativus* GlcAT14A. FvGT34 = *Fragaria vesca* MUCI10. SlXXT1 = *Solanum lycopersicum* XXT1. FvDUF288 = *Fragaria vesca* STELLO1.
(DOCX)

S2 Fig Comparison of detergent and sonication mediated cell lysis showed no solubilizing effect of detergent. Western blots against a cloning scar (α-attB2) of soluble lysis fractions resulting from either incubation and shaking with Triton-X100 or sonication. The left blot is from an *E*. *coli* culture overexpressing *Arabidopsis thaliana* MUR3 Δ1–100 (64 kDa), while the right is an *Arabidopsis thaliana* At1g53290 (45 kDa). These examples show that Triton-X100 is not solubilizing otherwise insoluble target material.
(DOCX)

S3 Fig SDS-PAGE of TEV cleavage of thioredoxin-XXT1 fusion protein. (DOCX)

S4 Fig Phylogenetic tree. A phylogenetic tree of the *in silico* analysis query sequences. Clustal Omega [30] was used to align the *Arabidopsis thaliana* CWGT protein sequences and produce a neighbour-joining tree. Ten groups were manually selected from the cladogram as indicated. Evolutionary distances are shown to the right of the gene ID.
(PDF)

S1 Table The test library of *Arabidopsis thaliana* CWGT sequences. (DOCX)

S2 Table Shortlist of interesting *Arabidopsis thaliana* CWGTs that formed the starting point for *in silico* target selection. (DOCX)

S3 Table Yields of the samples selected from the test library Labchip GXII analysis. Alphabetical list of the *Arabidopsis thaliana* CWGTs from the test library for which the automated capillary electrophoresis software detected a species of the expected molecular weight. The expression conditions are reported together with the yield calculated by the Labchip GXII software. No yield cutoff was applied in this initial pipeline validation experiment.
(DOCX)

S4 Table The library of non-*Arabidopsis* CWGT sequences. (DOCX)

S5 Table Yields and purities of the samples selected from the non-*Arabidopsis* library Labchip GXII analysis. Non-*Arabidopsis* CWGTs selected from the automated capillary electrophoresis results for SDS-PAGE, reported in the order of Fig 3 for easy referral. Samples were selected on the basis of a yield above μg/mL, a purity above 27%, or by visual inspection of the results in ‘gel mode’ (starred). Truncations and expression vectors are reported together with the yield and purity calculated by the Labchip GXII software. The well number is given in parenthesis after the protein name, to facilitate referral to Fig 3, and a unique identifier refers to S4 Table.
(DOCX)

S1 Data Automated capillary electrophoresis, SDS-PAGE, and mass spectrometry results for the test library screening. (XLSX)

S2 Data SDS-PAGE of selected samples from the test library screening, annotated with bands that where excised for mass spectrometry. (PPTX)

S3 Data Mass spectrometry data of samples selected from the test library screening. (SF3)

S4 Data Mass spectrometry data of samples selected from the test library screening. (SF3)

S5 Data Mass spectrometry data of samples selected from the non-*Arabidopsis* library screening. (SF3)

S6 Data Automated capillary electrophoresis, SDS-PAGE, and mass spectrometry results for the non-*Arabidopsis* library screening. (XLSX)

S1 List The CWGT-customized lysis buffer screen. (DOCX)

S1 Pred Predictions of secondary structure elements, transmembrane domains, and disordered domains for the sequences in the non-*Arabidopsis* library. (TXT)
