# Supplementary material for: Plant cell wall glycosyltransferases: High-throughput recombinant expression screening and general requirements for these challenging enzymes
Source: PLoS One. 2017 Jun 9;12(6):e0177591. doi: 10.1371/journal.pone.0177591 (PMC5466300; doi:10.1371/journal.pone.0177591)
Supplement: S1 Fig — Coomassie-stained SDS-PAGE of soluble (S) and insoluble (I) lysis fractions from E. coli expression of 4 different CWGTs. M = marker. CsGT14 = Cucumis sativus GlcAT14A. FvGT34 = Fragaria vesca MUCI10. SlXXT1 = Solanum lycopersicum XXT1. FvDUF288 = Fragaria vesca STELLO1. (DOCX) [file pone.0177591.s001.docx]

**S1 Fig. Recombinant CWGTs are mainly found in the insoluble lysis fraction.** Coomassie-stained SDS-PAGE of soluble (S) and insoluble (I) lysis fractions from *E. coli* expression of 4 different CWGTs. M = marker. CsGT14 = *Cucumis sativus* GlcAT14A. FvGT34 = *Fragaria vesca* MUCI10. SlXXT1 = *Solanum lycopersicum* XXT1. FvDUF288 = *Fragaria vesca* STELLO1.

**
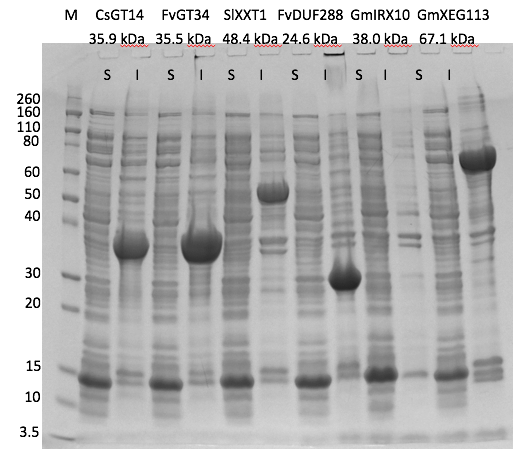
**

BL21(DE3) cells transformed with pET55DEST expression plasmid were cultured for 24 h at 18 ^o^C in LB-autoinduction media. At harvest, the cells were pelleted, then re-suspended in buffer (50mM HEPES, pH 8.0, 300mM NaCl, 5mM MgCl_2_, 20mM imidazole, 0.1mg/ml lysozyme, 0.3ug/ml DNaseI, 0.5mM Pefabloc (Sigma Aldrich, cat# 76307) protease inihibitor) and lysed by sonication with a microtip for 6 x 10 seconds. The lysate was clarified by centrifugation, the supernatant aspirated (soluble fraction, S) and the pellet re-suspended in an equal volume of buffer (insoluble fraction, I). Equal volumes of samples were run on 8-16% TG-SDS PAGE and stained with a colloidal coomassie blue dye (Instant Blue, Expedeon, cat# ISB1L).
