# Supplementary material for: Plant cell wall glycosyltransferases: High-throughput recombinant expression screening and general requirements for these challenging enzymes
Source: PLoS One. 2017 Jun 9;12(6):e0177591. doi: 10.1371/journal.pone.0177591 (PMC5466300; doi:10.1371/journal.pone.0177591)
Supplement: S2 Fig — Western blots against a cloning scar (α-attB2) of soluble lysis fractions resulting from either incubation and shaking with Triton-X100 or sonication. The left blot is from an E. coli culture overexpressing Arabidopsis thaliana MUR3 Δ1–100 (64 kDa), while the right is an Arabidopsis thaliana At1g53290 (45 kDa). These examples show that Triton-X100 is not solubilizing otherwise insoluble target material. (DOCX) [file pone.0177591.s002.docx]

**S2 Fig. Comparison of detergent and sonication mediated cell lysis showed no solubilizing effect of detergent.**

**Triton x-100 Sonication Triton X-100 Sonication**

*
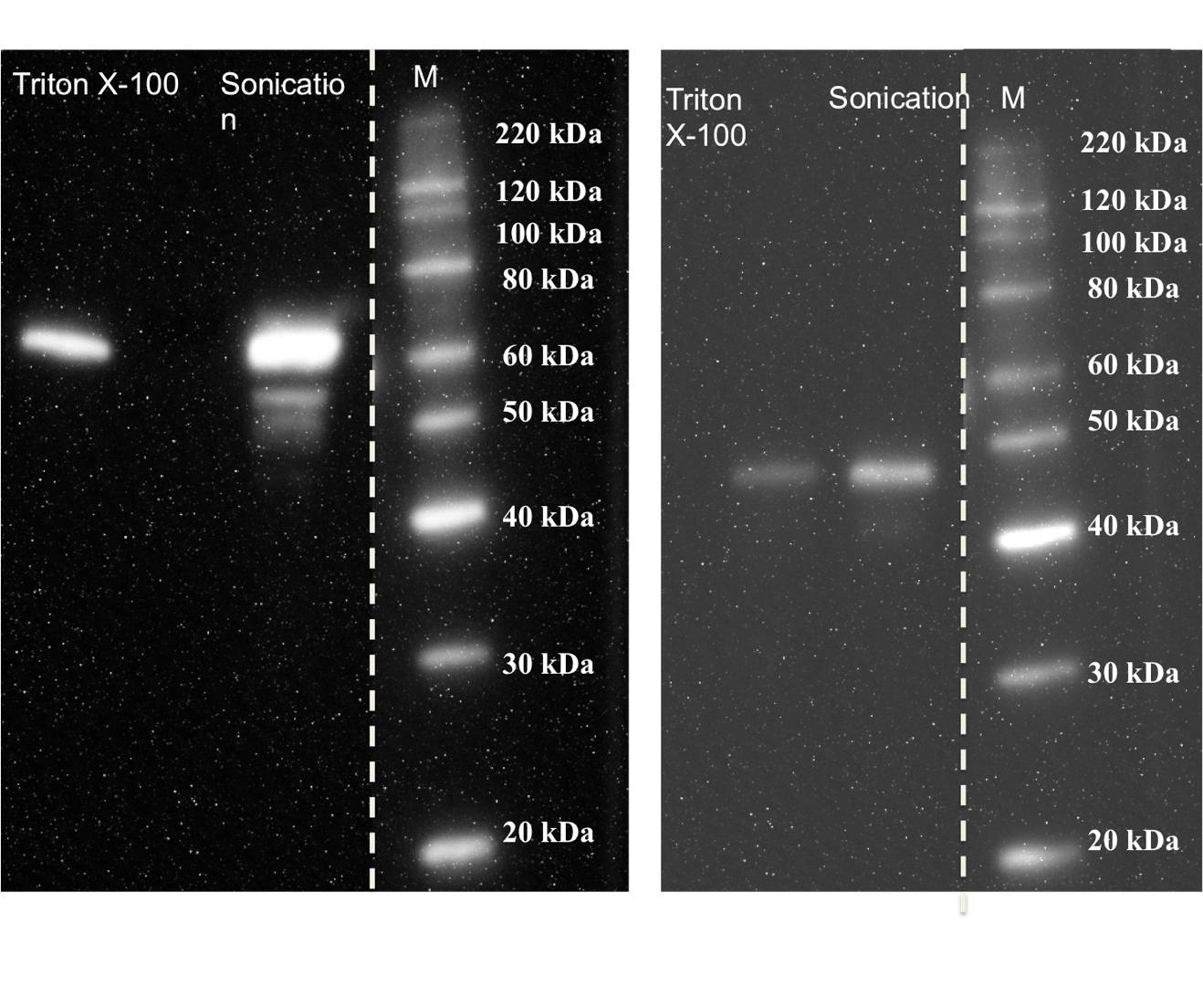
*

[α](https://en.wiktionary.org/wiki/%CE%B3%CE%BB%E1%BF%B6%CF%84%CF%84%CE%B1#Ancient_Greek)-attB2 western blots of soluble lysis fractions resulting from either incubation and shaking with Triton-X100 or sonication. The left blot is from an *E. coli* culture overexpressing *Arabidopsis thaliana* MUR3 Δ1-100 (64 kDa), while the right is a *Arabidopsis thaliana* At1g53290 (45 kDa). These examples show that Triton-X100 is not solubilizing otherwise insoluble target material.
