# Supplementary material for: Plant cell wall glycosyltransferases: High-throughput recombinant expression screening and general requirements for these challenging enzymes
Source: PLoS One. 2017 Jun 9;12(6):e0177591. doi: 10.1371/journal.pone.0177591 (PMC5466300; doi:10.1371/journal.pone.0177591)
Supplement: S3 Fig — (DOCX) [file pone.0177591.s003.docx]

**S3 Fig. SDS-PAGE of TEV cleavage of thioredoxin-XXT1 fusion protein.**


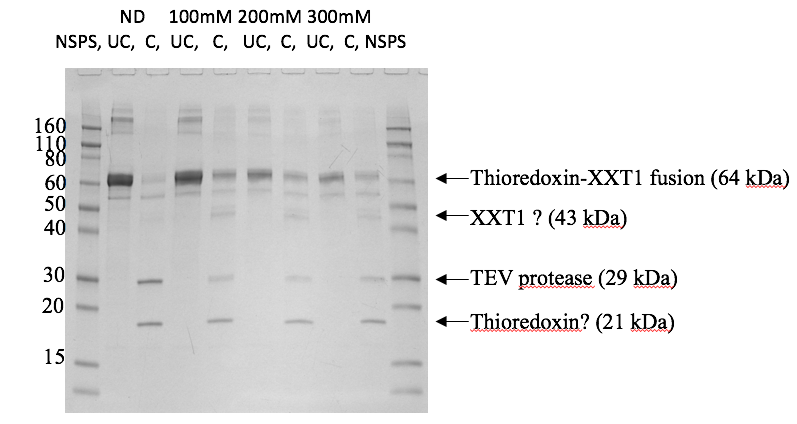


Coomassie-stained SDS-PAGE showing a sample of *Arabidopsis thaliana* XXT1(Δ1-50, Δ405-460) expressed as a thioredoxin fusion (His-thioredoxin-TEV site-XXT1) in the pET32dest vector, purified on nickel affinity chromatography and buffer exchanged to 25 mM HEPES pH 8.0, 300 mM NaCl, 5 mM MgCl_2_. The sample was aliquoted in 4 tubes, and not dialyzed (ND), or dialyzed against 25 mM Tris pH 8.0, 5 mM MgCl_2_ and varying amounts of NaCl overnight in the absence (UC=uncut) or presence (C=cut) of Tobacco Etch Virus (TEV) protease. Equal volumes of sample were loaded onto the gel after overnight incubation and centrifugation. The fusion protein is only stable when not dialyzed, or dialyzed against 25 mM Tris pH 8.0, 5 mM MgCl_2_, 100 mM NaCl. The mechanism behind this observation cannot be deduced from the present experiment but it makes those two conditions easier to interpret in terms of cleavage efficiency than the others. Addition of TEV protease yields in all cases only a very weak – and double-banded – species of the expected 43 kDa size. All samples contained visible precipitation after overnight incubation, and hence the very weak 43 kDa band is interpreted as XXT1 precipitating significantly upon cleavage. NSPS=Novex Sharp Pre-stained protein Standard (ThermoFisher Scientific, cat# LC5800).
