# Supplementary figures and images for: Plant cell wall glycosyltransferases: High-throughput recombinant expression screening and general requirements for these challenging enzymes
Source: PLoS One. 2017 Jun 9;12(6):e0177591. doi: 10.1371/journal.pone.0177591 (PMC5466300; doi:10.1371/journal.pone.0177591)

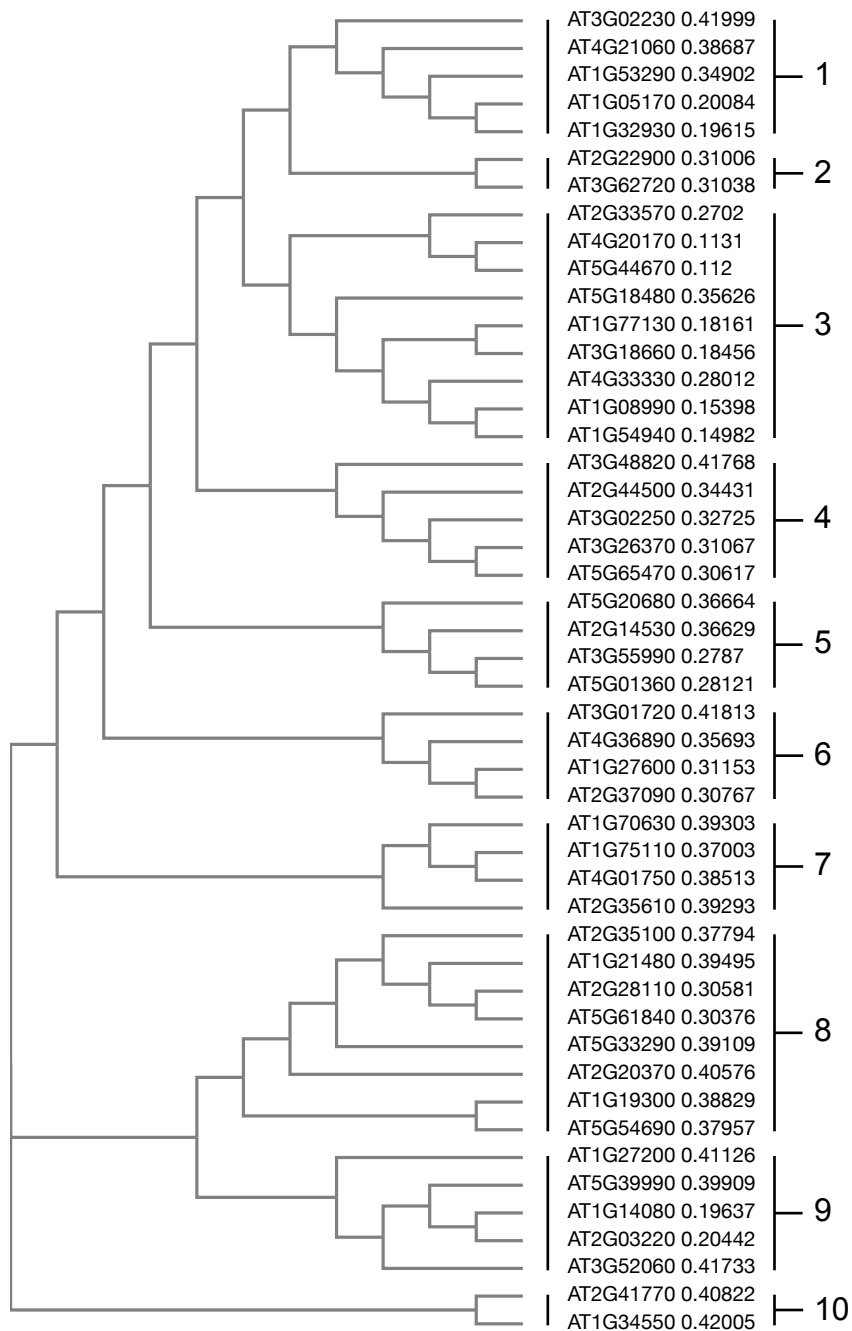

Supplement: S4 Fig — A phylogenetic tree of the in silico analysis query sequences. Clustal Omega [30] was used to align the Arabidopsis thaliana CWGT protein sequences and produce a neighbour-joining tree. Ten groups were manually selected from the cladogram as indicated. Evolutionary distances are shown to the right of the gene ID. (PDF) [file pone.0177591.s004.pdf]
