# Supplementary material for: Plant cell wall glycosyltransferases: High-throughput recombinant expression screening and general requirements for these challenging enzymes
Source: PLoS One. 2017 Jun 9;12(6):e0177591. doi: 10.1371/journal.pone.0177591 (PMC5466300; doi:10.1371/journal.pone.0177591)
Supplement: S1 Table — (DOCX) [file pone.0177591.s005.docx]

**S1 Table. The test library of *Arabidopsis thaliana* CWGT sequences.**

| **Locus** | **CAZy family** | **Protein name** | **Truncation** |
| --- | --- | --- | --- |
| At1g14080 | GT37 | FUT6 | none |
| At1g14080 | GT37 | FUT6 | Δ1-12 and Δ507-519 |
| At1g19300 | GT8 | Parvus | none |
| At1g19300 | GT8 | Parvus | Δ1-39 |
| At1g27600 | GT43 | IRX9L | none |
| At1g32930 | GT31 | Galt31A | none |
| At1g32930 | GT31 | Galt31A | Δ1-50 |
| At1g53290 | GT31 | none | none |
| At1g53290 | GT31 | none | Δ1-50 |
| At1g75110 | GT77 | RRA2 | none |
| At1g75110 | GT77 | RRA2 | Δ1-138 |
| At2g20370 | GT47 | MUR3 | none |
| At2g20370 | GT47 | MUR3 | Δ1-100 |
| At2g28110 | GT47 | IRX7 | none |
| At2g28110 | GT47 | IRX7 | Δ1-65 |
| At2g33570 | GT92 | GALS1 | none |
| At2g35100 | GT47 | ARAD1 | none |
| At2g35100 | GT47 | ARAD1 | Δ1-40 |
| At2g37090 | GT43 | IRX9  99 | none |
| At2g37090 | GT43 | IRX9 | Δ1-72 |
| At2g38650 | GT8 | GAUT7 | none |
| At3g02230 | GT75 | RGP1 | none |
| At3g18660 | GT8 | GUX1 | none |
| At3g18660 | GT8 | GUX1 | Δ1-100 and Δ631-659 |
| At3g48820 | GT29 | MGD2 | none |
| At3g48820 | GT29 | MGD2 | Δ1-120 |
| At3g61130 | GT8 | GAUT1 | none |
| At3g62720 | GT34 | XXT1 | none |
| At3g62720 | GT34 | XXT1 | Δ1-50 and Δ405-460 |
| At4g01750 | GT77 | RGXT2 | none |
| At4g01750 | GT77 | RGXT2 | Δ1-100 |
| At4g36890 | GT43 | IRX14 | None |
| At4g36890 | GT43 | IRX14  14 | Δ1-60 |
| At5g44930 | GT47 | ARAD2 | none |
| At5g54690 | GT8 | IRX8 | none |
| At5g54690 | GT8 | IRX8 | Δ1-70 |
| At5g61840 | GT47 | GUT1/IRX10L | none |
| At5g61840 | GT47 | GUT1/IRX10L | Δ1-46 |
