# Supplementary material for: Plant cell wall glycosyltransferases: High-throughput recombinant expression screening and general requirements for these challenging enzymes
Source: PLoS One. 2017 Jun 9;12(6):e0177591. doi: 10.1371/journal.pone.0177591 (PMC5466300; doi:10.1371/journal.pone.0177591)
Supplement: S2 Table — (DOCX) [file pone.0177591.s006.docx]

**S2 Table. Shortlist of interesting *Arabidopsis thaliana* CWGTs that formed the starting point for *in silico* target selection.**

| **protein** | **family** | **locus** |
| --- | --- | --- |
| ARAD1 | GT47 | At2g35100 |
| FUT1 | GT37 | At2g03220 |
| FUT6 | GT37 | At1g14080 |
| GALS1 | GT92 | At2g33570 |
| GALS2 | GT92 | At5g44670 |
| GALS3 | GT92 | At4g20170 |
| GalT2 | GT31 | At4g21060 |
| Galt31A | GT31 | At1g32930 |
| GlcAT14A | GT14 | At5g39990 |
| MUCI10 | GT34 | At2g22900 |
| GUX1 | GT8 | At3g18660 |
| GUX2 | GT8 | At4g33330 |
| GUX3 | GT8 | At1g77130 |
| GUX4 | GT8 | At1g54940 |
| GUX5 | GT8 | At1g08990 |
| IPUT1 | GT8 | At5g18480 |
| GUT1/IRX10L | GT47 | At5g61840 |
| Irx14 | GT43 | At4g36890 |
| IRX7 | GT47 | At2g28110 |
| IRX8 | GT8 | At5g54690 |
| Irx9 | GT43 | At2g37090 |
| IRX9L | GT43 | At1g27600 |
| MGD2 | GT29 | At3g48820 |
| MUR3 | GT47 | At2g20370 |
| NN | GT31 | At1g05170 |
| NN | GT47 | At1g21480 |
| NN | DUF23 | At1g27200 |
| NN | DUF616 | At1g34550 |
| NN | GT31 | At1g53290 |
| NN | DUF288 | At2g41770 |
| NN | DUF246 | At2g44500 |
| NN | DUF246 | At3g02250 |
| NN | DUF246 | At3g26370 |
| NN | DUF246 | At5g65470 |
| Parvus | GT8 | At1g19300 |
| RAY1 | GT77 | At1g70630 |
| RGP1 | GT75 | At3g02230 |
| RGXT2 | GT77 | At4g01750 |
| RRA2 | GT77 | At1g75110 |
| SGT1 | GT96 | At3g01720 |
| XEG113 | GT77 | At2g35610 |
| XGD1 | GT47 | At5g33290 |
| XXT1 | GT34 | At3g62720 |
