# Supplementary material for: Plant cell wall glycosyltransferases: High-throughput recombinant expression screening and general requirements for these challenging enzymes
Source: PLoS One. 2017 Jun 9;12(6):e0177591. doi: 10.1371/journal.pone.0177591 (PMC5466300; doi:10.1371/journal.pone.0177591)
Supplement: S3 Table — Alphabetical list of the Arabidopsis thaliana CWGTs from the test library for which the automated capillary electrophoresis software detected a species of the expected molecular weight. The expression conditions are reported together with the yield calculated by the Labchip GXII software. No yield cutoff was applied in this initial pipeline validation experiment. (DOCX) [file pone.0177591.s007.docx]

**S3 Table. Yields of the samples selected from the test library Labchip GXII analysis.**

| **Protein** | **Construct** | **Vector** | **Chaperone co-expression** | **Mw (kDa)** | **Yield (**µ**g/mL)** |
| --- | --- | --- | --- | --- | --- |
| ARAD1 | full-length | pET55dest | none | 55.5 | 15.6 |
| ARAD1 | full-length | pET55dest | GroEL. GroES | 55.5 | 2.6 |
| ARAD1 | full-length | pET55dest | Trigger factor | 55.5 | 1.1 |
| ARAD1 | Δ1-40 | pET55dest | none | 51.3 | 35.2 |
| ARAD1 | Δ1-40 | pET55dest | GroEL. GroES | 51.3 | 3.1 |
| ARAD1 | Δ1-40 | pET55dest | Trigger factor | 51.3 | 0.6 |
| ARAD2 | full-length | pET55dest | none | 55.7 | 24.4 |
| ARAD2 | full-length | pET32dest | GroEL. GroES | 77.2 | 7.0 |
| ARAD2 | full-length | pET55dest | GroEL. GroES | 55.7 | 3.5 |
| ARAD2 | full-length | pET32dest | DnaK. DnaJ. GrpE | 77.2 | 0.8 |
| At1g53290 | full-length | pET55dest | none | 43.5 | 30.7 |
| At1g53290 | full-length | pET55dest | DnaK. DnaJ. GrpE | 43.5 | 18.0 |
| At1g53290 | full-length | pET55dest | GroEL. GroES | 43.5 | 13.7 |
| At1g53290 | full-length | pET32dest | Trigger factor | 65.0 | 0.4 |
| At1g53290 | Δ1-50 | pET55dest | none | 38.2 | 60.9 |
| At1g53290 | Δ1-50 | pET55dest | GroEL. GroES | 38.2 | 25.7 |
| At1g53290 | Δ1-50 | pET55dest | DnaK. DnaJ. GrpE | 38.2 | 5.3 |
| At1g53290 | Δ1-50 | pET32dest | DnaK. DnaJ. GrpE | 59.7 | 1.2 |
| FUT6 | full-length | pET32dest | GroEL. GroES | 85.5 | 7.1 |
| FUT6 | full-length | pET55dest | none | 64.0 | 0.9 |
| FUT6 | full-length | pET32dest | GroEL. GroES. DnaK. DnaJ. GrpE | 85.5 | 0.4 |
| FUT6 | Δ1-12 and Δ507-519 | pET55dest | none | 58.0 | 11.7 |
| FUT6 | Δ1-12 and Δ507-519 | pET55dest | GroEL. GroES | 58.0 | 5.5 |
| FUT6 | Δ1-12 and Δ507-519 | pET22dest | Trigger factor | 59.1 | 1.8 |
| GALS1 | full-length | pET55dest | none | 61.6 | 14.1 |
| GALS1 | full-length | pET55dest | GroEL. GroES | 61.6 | 11.8 |
| GALS1 | full-length | pET32dest | GroEL. GroES | 83.1 | 7.7 |
| GALS1 | full-length | pET32dest | GroEL. GroES. DnaK. DnaJ. GrpE | 83.1 | 0.6 |
| GALS1 | full-length | pET22dest | Trigger factor | 62.7 | 0.4 |
| Galt31A | full-length | pET55dest | DnaK. DnaJ. GrpE | 49.3 | 127.4 |
| Galt31A | full-length | pET55dest | none | 49.3 | 14.6 |
| Galt31A | full-length | pET55dest | GroEL. GroES | 49.3 | 13.1 |
| Galt31A | full-length | pET32dest | Trigger factor | 70.8 | 0.6 |
| Galt31A | full-length | pET32dest | DnaK. DnaJ. GrpE | 70.8 | 0.1 |
| Galt31A | Δ1-50 | pET55dest | none | 43.9 | 27.3 |
| Galt31A | Δ1-50 | pET55dest | GroEL. GroES | 43.9 | 10.8 |
| GAUT1 | full-length | pET55dest | GroEL. GroES | 82.1 | 53.1 |
| GAUT1 | full-length | pET55dest | none | 82.1 | 4.0 |
| GAUT7 | full-length | pET55dest | GroEL. GroES | 74.4 | 53.0 |
| GAUT7 | full-length | pET55dest | none | 74.4 | 5.1 |
| GAUT7 | full-length | pET55dest | Trigger factor | 74.4 | 1.1 |
| GUT1/IRX10L | full-length | pET55dest | GroEL. GroES | 51.9 | 12.2 |
| GUT1/IRX10L | full-length | pET55dest | none | 51.9 | 10.1 |
| GUT1/IRX10L | full-length | pET32dest | Trigger factor | 73.4 | 1.7 |
| GUT1/IRX10L | full-length | pET22dest | GroEL. GroES | 53.0 | 0.7 |
| GUT1/IRX10L | full-length | pET55dest | Trigger factor | 51.9 | 0.1 |
| GUT1/IRX10L | Δ1-46 | pET55dest | none | 46.9 | 50.0 |
| GUT1/IRX10L | Δ1-46 | pET55dest | GroEL. GroES | 46.9 | 9.6 |
| GUT1/IRX10L | Δ1-46 | pET55dest | GroEL. GroES. DnaK. DnaJ. GrpE | 46.9 | 3.6 |
| GUT1/IRX10L | Δ1-46 | pET22dest | Trigger factor | 48.0 | 1.9 |
| GUT1/IRX10L | Δ1-46 | pET32dest | Trigger factor | 68.4 | 1.0 |
| GUT1/IRX10L | Δ1-46 | pET55dest | DnaK. DnaJ. GrpE | 46.9 | 0.6 |
| GUT1/IRX10L | Δ1-46 | pET55dest | Trigger factor | 46.9 | 0.3 |
| GUX1 | full-length | pET55dest | GroEL. GroES | 81.0 | 13.1 |
| GUX1 | full-length | pET55dest | none | 81.0 | 4.6 |
| GUX1 | full-length | pET32dest | DnaK. DnaJ. GrpE | 102.5 | 0.4 |
| GUX1 | Δ1-100 and Δ631-659 | pET55dest | none | 66.8 | 11.0 |
| GUX1 | Δ1-100 and Δ631-659 | pET55dest | GroEL. GroES | 66.8 | 2.5 |
| GUX1 | Δ1-100 and Δ631-659 | pET55dest | Trigger factor | 66.8 | 0.2 |
| IRX14 | full-length | pET32dest | GroEL. GroES | 85.3 | 4.9 |
| IRX14 | full-length | pET55dest | GroEL. GroES | 63.8 | 2.0 |
| IRX14 | full-length | pET32dest | DnaK. DnaJ. GrpE | 85.3 | 0.4 |
| IRX14 | Δ1-60 | pET32dest | GroEL. GroES | 78.6 | 7.5 |
| IRX14 | Δ1-60 | pET55dest | none | 57.1 | 4.7 |
| IRX14 | Δ1-60 | pET55dest | GroEL. GroES | 57.1 | 1.2 |
| IRX14 | Δ1-60 | pET55dest | Trigger factor | 57.1 | 0.5 |
| IRX7 | full-length | pET55dest | none | 56.4 | 16.8 |
| IRX7 | full-length | pET55dest | GroEL. GroES | 56.4 | 3.2 |
| IRX7 | full-length | pET32dest | GroEL. GroES | 77.9 | 0.8 |
| IRX7 | full-length | pET22dest | Trigger factor | 57.5 | 0.2 |
| IRX8 | full-length | pET55dest | GroEL. GroES | 65.6 | 2.7 |
| IRX8 | full-length | pET22dest | GroEL. GroES. DnaK. DnaJ. GrpE | 66.7 | 0.1 |
| IRX9 | full-length | pET55dest | GroEL. GroES | 44.8 | 13.6 |
| IRX9 | full-length | pET55dest | none | 44.8 | 8.0 |
| IRX9 | full-length | pET32dest | DnaK. DnaJ. GrpE | 66.3 | 4.6 |
| IRX9 | full-length | pET32dest | Trigger factor | 66.3 | 0.8 |
| IRX9 | Δ1-72 | pET55dest | GroEL. GroES | 36.7 | 24.1 |
| IRX9 | Δ1-72 | pET55dest | none | 36.7 | 9.4 |
| IRX9 | Δ1-72 | pET55dest | Trigger factor | 36.7 | 7.0 |
| IRX9 | Δ1-72 | pET55dest | GroEL. GroES | 36.7 | 3.8 |
| IRX9 | Δ1-72 | pET55dest | none | 36.7 | 3.1 |
| IRX9 | Δ1-72 | pET32dest | DnaK. DnaJ. GrpE | 58.2 | 0.3 |
| IRX9 | Δ1-72 | pET55dest | DnaK. DnaJ. GrpE | 36.7 | 0.2 |
| IRX9L | full-length | pET55dest | none | 49.9 | 9.9 |
| IRX9L | full-length | pET55dest | GroEL. GroES | 49.9 | 6.4 |
| MGD2 | full-length | pET55dest | none | 54.2 | 7.9 |
| MGD2 | full-length | pET55dest | GroEL. GroES | 54.2 | 1.6 |
| MGD2 | full-length | pET32dest | DnaK. DnaJ. GrpE | 75.7 | 0.1 |
| MGD2 | Δ1-120 | pET55dest | none | 40.9 | 26.2 |
| MGD2 | Δ1-120 | pET55dest | GroEL. GroES. DnaK. DnaJ. GrpE | 40.9 | 5.7 |
| MGD2 | Δ1-120 | pET55dest | GroEL. GroES | 40.9 | 3.9 |
| MGD2 | Δ1-120 | pET55dest | DnaK. DnaJ. GrpE | 40.9 | 2.8 |
| MGD2 | Δ1-120 | pET55dest | Trigger factor | 40.9 | 1.1 |
| MUR3 | full-length | pET55dest | GroEL. GroES | 75.4 | 118.9 |
| MUR3 | full-length | pET32dest | DnaK. DnaJ. GrpE | 96.9 | 24.4 |
| MUR3 | full-length | pET55dest | none | 75.4 | 8.1 |
| MUR3 | full-length | pET55dest | GroEL. GroES. DnaK. DnaJ. GrpE | 75.4 | 3.0 |
| MUR3 | full-length | pET55dest | Trigger factor | 75.4 | 1.0 |
| MUR3 | Δ1-100 | pET55dest | GroEL. GroES | 64.2 | 117.9 |
| MUR3 | Δ1-100 | pET55dest | none | 64.2 | 12.9 |
| MUR3 | Δ1-100 | pET55dest | DnaK. DnaJ. GrpE | 64.2 | 6.8 |
| MUR3 | Δ1-100 | pET22dest | Trigger factor | 65.3 | 1.7 |
| MUR3 | Δ1-100 | pET32dest | GroEL. GroES. DnaK. DnaJ. GrpE | 85.7 | 0.3 |
| Parvus | full-length | pET55dest | GroEL. GroES | 43.7 | 11.0 |
| Parvus | full-length | pET55dest | none | 43.7 | 9.5 |
| Parvus | Δ1-39 | pET55dest | none | 39.3 | 16.4 |
| Parvus | Δ1-39 | pET22dest | DnaK. DnaJ. GrpE | 40.4 | 13.0 |
| Parvus | Δ1-39 | pET55dest | Trigger factor | 39.3 | 1.6 |
| Parvus | Δ1-39 | pET22dest | GroEL. GroES. DnaK. DnaJ. GrpE | 40.4 | 1.1 |
| Parvus | Δ1-39 | pET22dest | none | 40.4 | 0.9 |
| Parvus | Δ1-39 | pET55dest | GroEL. GroES. DnaK. DnaJ. GrpE | 39.3 | 0.8 |
| RGP1 | full-length | pET55dest | GroEL. GroES | 45.3 | 271.8 |
| RGP1 | full-length | pET55dest | none | 45.3 | 111.0 |
| RGP1 | full-length | pET55dest | Trigger factor | 45.3 | 1.6 |
| RGP1 | full-length | pET32dest | DnaK. DnaJ. GrpE | 66.8 | 1.6 |
| RGP1 | full-length | pET32dest | Trigger factor | 66.8 | 1.3 |
| RGP1 | full-length | pET22dest | none | 46.4 | 0.5 |
| RGP1 | full-length | pET55dest | DnaK. DnaJ. GrpE | 45.3 | 0.5 |
| RGP1 | full-length | pET22dest | Trigger factor | 46.4 | 0.4 |
| RGXT2 | full-length | pET55dest | none | 46.5 | 14.5 |
| RGXT2 | full-length | pET55dest | GroEL. GroES | 46.5 | 2.1 |
| RGXT2 | full-length | pET22dest | DnaK. DnaJ. GrpE | 47.6 | 0.1 |
| RGXT2 | Δ1-100 | pET55dest | none | 37.0 | 22.0 |
| RGXT2 | Δ1-100 | pET55dest | GroEL. GroES | 37.0 | 3.3 |
| RGXT2 | Δ1-100 | pET22dest | GroEL. GroES | 38.1 | 0.4 |
| RGXT2 | Δ1-100 | pET32dest | none | 58.5 | 0.4 |
| RGXT2 | Δ1-100 | pET22dest | none | 38.1 | 0.3 |
| RRA2 | full-length | pET55dest | none | 52.9 | 5.7 |
| RRA2 | full-length | pET55dest | GroEL. GroES | 52.9 | 2.8 |
| RRA2 | Δ1-138 | pET55dest | GroEL. GroES | 37.8 | 4.0 |
| RRA2 | Δ1-138 | pET55dest | none | 37.8 | 4.0 |
| RRA2 | Δ1-138 | pET32dest | GroEL. GroES. DnaK. DnaJ. GrpE | 59.3 | 0.4 |
| RRA2 | Δ1-138 | pET55dest | Trigger factor | 37.8 | 0.2 |
| XXT1 | full-length | pET55dest | none | 58.1 | 13.5 |
| XXT1 | full-length | pET32dest | DnaK. DnaJ. GrpE | 79.6 | 10.9 |
| XXT1 | full-length | pET32dest | GroEL. GroES | 79.6 | 9.7 |
| XXT1 | full-length | pET32dest | Trigger factor | 79.6 | 9.6 |
| XXT1 | full-length | pET55dest | GroEL. GroES | 58.1 | 8.5 |
| XXT1 | full-length | pET55dest | Trigger factor | 58.1 | 1.2 |
| XXT1 | full-length | pET32dest | GroEL. GroES. DnaK. DnaJ. GrpE | 79.6 | 0.8 |
| XXT1 | full-length | pET22dest | Trigger factor | 59.2 | 0.3 |
| XXT1 | Δ1-50 and Δ405-460 | pET32dest | GroEL. GroES | 67.9 | 47.0 |
| XXT1 | Δ1-50 and Δ405-460 | pET55dest | none | 46.4 | 23.3 |
| XXT1 | Δ1-50 and Δ405-460 | pET55dest | GroEL. GroES | 46.4 | 17.3 |
| XXT1 | Δ1-50 and Δ405-460 | pET32dest | Trigger factor | 67.9 | 15.0 |
| XXT1 | Δ1-50 and Δ405-460 | pET55dest | Trigger factor | 46.4 | 14.9 |
| XXT1 | Δ1-50 and Δ405-460 | pET32dest | DnaK. DnaJ. GrpE | 67.9 | 1.5 |
| XXT1 | Δ1-50 and Δ405-460 | pET22dest | GroEL. GroES | 47.5 | 0.4 |

Alphabetical list of the *Arabidopsis thaliana* CWGTs from the test library for which the automated capillary electrophoresis software detected a species of the expected molecular weight. The expression conditions are reported together with the yield calculated by the Labchip GXII software. No yield cutoff was applied in this initial pipeline validation experiment.
