# Supplementary material for: Plant cell wall glycosyltransferases: High-throughput recombinant expression screening and general requirements for these challenging enzymes
Source: PLoS One. 2017 Jun 9;12(6):e0177591. doi: 10.1371/journal.pone.0177591 (PMC5466300; doi:10.1371/journal.pone.0177591)
Supplement: S4 Table — (DOCX) [file pone.0177591.s008.docx]

| **S4 Table. The library of non-*Arabidopsis* CWGT sequences.** | | | | | | | |
| --- | --- | --- | --- | --- | --- | --- | --- |
| **ID** | **locus** | **source organism** | **CAZy** | **parent protein name** | **construct (FL = full-length)** | **cysteines** | **glycosylation sites** |
| D01_FL | 100836426 | *Brachypodion distachyon* | GT75 | RGP1 | 1-363 (FL) | 7 | 0 |
| D02_FL | 100282614 | *Zea mays* | GT75 | RGP1 | 1-361 (FL) | 7 | 0 |
| D02_Clone01 |  |  |  |  | 1-134 |  |  |
| D02_Clone02 |  |  |  |  | 1-236 |  |  |
| D02_Clone03 |  |  |  |  | 1-346 |  |  |
| D03_FL | 101262652 | *Solanum lycopersicum* | GT34 | XXT1 | 1-464 (FL) | 6 | 1 |
| D03_Clone01 |  |  |  |  | 47-464 |  |  |
| D04_FL | 101291758 | *Fragaria vesca* | GT34 | MUCI10 | 1-457 (FL) | 5 | 1 |
| D04_Clone01 |  |  |  |  | 92-457 |  |  |
| D04_Clone02 |  |  |  |  | 58-457 |  |  |
| D04_Clone03 |  |  |  |  | 92-392 |  |  |
| D04_Clone04 |  |  |  |  | 58-392 |  |  |
| D05_FL | 4350696 | *Oryza sativa* | GT34 | MUCI10 | 1-483 (FL) | 6 | 1 |
| D05_Clone01 |  |  |  |  | 73-483 |  |  |
| D05_Clone02 |  |  |  |  | 105-483 |  |  |
| D05_Clone03 |  |  |  |  | 73-429 |  |  |
| D05_Clone04 |  |  |  |  | 105-429 |  |  |
| D06_FL | 100787815 | *Glycine max* | GT92 | GALS1 | 1-515 (FL) | 8 | 4 |
| D06_Clone01 |  |  |  |  | 109-515 |  |  |
| D06_Clone02 |  |  |  |  | 50-515 |  |  |
| D07_FL | 101311123 | *Fragaria vesca* | n/a | DUF246 | 1-577 (FL) | 7 | 1 |
| D07_Clone01 |  |  |  |  | 92-577 |  |  |
| D08_FL | 101776477 | *Setaria italica* | n/a | DUF246 | 1-499 (FL) | 8 | 2 |
| D08_Clone01 |  |  |  |  | 64-499 |  |  |
| D09_FL | 101299994 | *Fragaria vesca* | n/a | DUF246 | 1-475 (FL) | 5 | 2 |
| D09_Clone01 |  |  |  |  | 47-475 |  |  |
| D10_FL | 100802467 | *Glycine max* | n/a | TBL13 | 1-410 (FL) | 9 | 2 |
| D10_Clone01 |  |  |  |  | 65-410 |  |  |
| D10_Clone02 |  |  |  |  | 65-374 |  |  |
| D11_FL | 100825801 | *Brachypodion distachyon* | n/a | TBL29 | 1-539 (FL) | 8 | 3 |
| D11_Clone01 |  |  |  |  | 88-539 |  |  |
| D12_FL | 606306 | *Hordeum vulgare* | GT43 | IRX9L | 1-393 (FL) | 4 | 2 |
| D12_Clone01 |  |  |  |  | 95-393 |  |  |
| D13_FL | 100777505 | *Glycine max* | GT43 | IRX14 | 1-502 (FL) | 4 | 2 |
| D13_Clone01 |  |  |  |  | 79-502 |  |  |
| D13_Clone02 |  |  |  |  | 157-502 |  |  |
| D13_Clone03 |  |  |  |  | 79-435 |  |  |
| D13_Clone04 |  |  |  |  | 157-435 |  |  |
| D14_FL | 101228909 | *Cucumis sativus* | GT8 | PARVUS | 1-283 (FL) | 8 | 1 |
| D15_FL | 4336486 | *Oryza sativa* | GT8 | PARVUS | 1-316 (FL) | 4 | 0 |
| D15_Clone01 |  |  |  |  | 39-316 |  |  |
| D16_FL | 100783737 | *Glycine max* | GT47 | GUT1/IRX10L | 1-487 (FL) | 6 | 1 |
| D16_Clone01 |  |  |  |  | 43-497 |  |  |
| D17_FL | 100794632 | *Glycine max* | GT47 | GUT1/IRX10L | 1-506 (FL) | 6 | 1 |
| D17_Clone01 |  |  |  |  | 43-506 |  |  |
| D17_Clone02 |  |  |  |  | 175-506 |  |  |
| D17_Clone03 |  |  |  |  | 43-439 |  |  |
| D17_Clone04 |  |  |  |  | 175-506 |  |  |
| D18_FL | 101210575 | *Cucumis sativus* | GT14 | GlcAT14A | 1-396 (FL) | 5 | 1 |
| D18_Clone01 |  |  |  |  | 30-396 |  |  |
| D18_Clone02 |  |  |  |  | 57-396 |  |  |
| D18_Clone03 |  |  |  |  | 30-342 |  |  |
| D18_Clone04 |  |  |  |  | 57-342 |  |  |
| D19_FL | 101510562 | *Cicer arietinum* | n/a | DUF288 | 1-771 (FL) | 3 | 2 |
| D19_Clone01 |  |  |  |  | 115-771 |  |  |
| D20_FL | 100797815 | *Glycine max* | n/a | EMB2756 | 1-699 (FL) | 8 | 4 |
| D20_Clone01 |  |  |  |  | 105-699 |  |  |
| D21_FL | 101253530 | *Solanum lycopersicum* | n/a | DUF288 | 1-760 (FL) | 2 | 2 |
| D21_Clone01 |  |  |  |  | 105-760 |  |  |
| D22_FL | 101309981 | *Fragaria vesca* | n/a | DUF288 | 1-761 (FL) | 2 | 2 |
| D22_Clone01 |  |  |  |  | 96-761 |  |  |
| D22_Clone02 |  |  |  |  | 96-252 |  |  |
| D22_Clone03 |  |  |  |  | 96-310 |  |  |
| D23_FL | 100255856 | *Vitis vinifera* | GT77 | RRA | 1-434 (FL) | 3 | 1 |
| D23_Clone01 |  |  |  |  | 54-434 |  |  |
| D23_Clone02 |  |  |  |  | 54-403 |  |  |
| D24_FL | 100819032 | *Glycine max* | GT77 | XEG113 | 1-639 (FL) | 9 | 1 |
| D24_Clone01 |  |  |  |  | 53-639 |  |  |
| D24_Clone02 |  |  |  |  | 120-639 |  |  |
