# Supplementary material for: Plant cell wall glycosyltransferases: High-throughput recombinant expression screening and general requirements for these challenging enzymes
Source: PLoS One. 2017 Jun 9;12(6):e0177591. doi: 10.1371/journal.pone.0177591 (PMC5466300; doi:10.1371/journal.pone.0177591)
Supplement: S5 Table — Non-Arabidopsis CWGTs selected from the automated capillary electrophoresis results for SDS-PAGE, reported in the order of Fig 3 for easy referral. Samples were selected on the basis of a yield above μg/mL, a purity above 27%, or by visual inspection of the results in ‘gel mode’ (starred). Truncations and expression vectors are reported together with the yield and purity calculated by the Labchip GXII software. The well number is given in parenthesis after the protein name, to facilitate referral to Fig 3, and a unique identifier refers to S4 Table. (DOCX) [file pone.0177591.s009.docx]

**S5 Table. Yields and purities of the samples selected from the non-*Arabidopsis* library Labchip GXII analysis.**

| **ID** | **Protein** | **Construct** | **Vector** | **Mw (kDa)** | **Yield (**µ**g/mL)** | **Purity (%)** |  |
| --- | --- | --- | --- | --- | --- | --- | --- |
| D02_Clone01 | ZmRGP1 (a4) | Δ135-361 | pET55dest | 20.1 | 30,3 | 14 | * |
| D24_Clone02 | GmXEG113 (a5) | Δ1-119 | pET55dest | 62.5 | 67,2 | 22 | * |
| D04_Clone01 | FvMUCI10 (a6) | Δ1-91 | pET55dest | 45.6 | 35,9 | 14 | * |
| D18_Clone03 | CsGlcAT14A (a9) | Δ1-29 and Δ343-396 | pET55dest | 39.7 | 86,3 | 52 |  |
| D10_FL | GmTBL13 (b2) | full-length | pET55dest | 52.4 | 125,4 | 21 |  |
| D02_Clone02 | ZmRGP1 (b4) | Δ237-361 | pET55dest | 31.4 | 23,5 | 22 | * |
| D08_Clone01 | SiDUF246 (b5) | Δ1-63 | pET55dest | 53.3 | 24,3 | 36 |  |
| D13_Clone03 | GmIRX14 (b7) | Δ1-78 and Δ436-502 | pET55dest | 44.6 | 119,8 | 46 |  |
| D18_Clone03 | CsGlcAT14A (b8) | Δ1-56 and Δ343-396 | pET55dest | 36.8 | 75,8 | 66 |  |
| D07_Clone01 | FvDUF26 (c5) | Δ1-63 | pET55dest | 58.8 | 21,5 | 32 |  |
| D04_Clone03 | FvMUCI10 (c6) | Δ1-91 and Δ393-457 | pET55dest | 38.4 | 120,6 | 36 |  |
| D22_Clone01 | FvDUF288 (c7) | Δ1-95 | pET55dest | 78.6 | 110,8 | 42 |  |
| D10_Clone01 | GmTBL13 (c8) | Δ1-64 | pET55dest | 43.4 | 45,5 | 33 |  |
| D03_Clone01 | SlXXT1 (c9) | Δ1-46 | pET55dest | 51.3 | 40,2 | 50 |  |
| D06_Clone01 | GmGALS1 (d5) | Δ1-108 | pET55dest | 50.1 | 66,4 | 19 | * |
| D04_Clone04 | FvMUCI10 (d6) | Δ1-57 and Δ393-457 | pET55dest | 42.1 | 223,5 | 47 |  |
| D22_Clone02 | FvDUF288 (d7) | Δ1-95 and Δ253-761 | pET55dest | 22.6 | 26,9 | 14 | * |
| D10_Clone02 | GmTBL13 (d8) | Δ1-64 and Δ375-410 | pET55dest | 39.4 | 23,2 | 28 |  |
| D19_Clone01 | CaDUF288 (d9) | Δ1-114 | pET55dest | 77.6 | 100,2 | 29 |  |
| D13_FL | GmIRX14 (e2) | full-length | pET55dest | 61.4 | 57,9 | 16 | * |
| D22_Clone03 | FvDUF288 (e7) | Δ1-95 and Δ311-761 | pET55dest | 28.9 | 45,9 | 51 |  |
| D06_Clone02 | GmGALS1 (e8) | Δ1-49 | pET55dest | 56.6 | 21,3 | 20 | * |
| D21_Clone01 | SlDUF288 (e9) | Δ1-104 | pET55dest | 77.5 | 56,2 | 59 |  |
| D13_Clone04 | GmIRX14 (f5) | Δ1-156 and Δ436-502 | pET55dest | 36.0 | 94,8 | 67 |  |
| D07_FL | FvDUF246 (g1) | full-length | pET55dest | 70.5 | 103,3 | 12 |  |
| D23_Clone01 | VvRRA (g5) | Δ1-53 | pET55dest | 47.2 | 63,3 | 30 |  |
| D20_Clone01 | GmEMB2756 (g6) | Δ1-104 | pET55dest | 70.7 | 20,0 | 18 | * |
| D17_Clone04 | GmGUT1/IRX10L (g8) | Δ1-174 and Δ440-506 | pET55dest | 34.4 | 103,5 | 7 |  |
| D24_Clone01 | GmXEG113 (h6) | Δ1-52 | pET55dest | 70.0 | 41,2 | 32 |  |
| D18_Clone01 | CsGlcAT14A (h8) | Δ1-29 | pET55dest | 45.7 | 77,2 | 31 |  |
| D02_Clone01 | ZmRGP1 (a4) | Δ135-361 | pET22dest | 20.4 | 41,5 | 44 |  |
| D13_Clone01 | GmIRX14 (a7) | Δ1-78 | pET22dest | 52.2 | 48,0 | 15 | * |
| D18_Clone02 | CsGlcAT14A (a8) | Δ1-56 | pET22dest | 43.0 | 74,6 | 20 |  |
| D02_Clone02 | ZmRGP1 (b4) | Δ135-361 | pET22dest | 31.7 | 42,0 | 25 | * |
| D08_Clone01 | SiDUF246 (b5) | Δ1-63 | pET22dest | 53.6 | 13,2 | 28 |  |
| D04_Clone02 | FvMUCI10 (b6) | Δ1-57 | pET22dest | 49.6 | 108,3 | 24 |  |
| D04_Clone03 | FvMUCI10 (c6) | Δ1-91 and Δ393-457 | pET22dest | 38.7 | 41,6 | 16 | * |
| D06_Clone01 | GmGALS1 (d5) | Δ1-108 | pET22dest | 50.4 | 33,7 | 25 | * |
| D04_Clone04 | FvMUCI10 (d6) | Δ1-57 and Δ393-457 | pET22dest | 42.4 | 9,3 | 3 | * |
| D22_Clone03 | FvDUF288 (e7) | Δ1-95 and Δ311-761 | pET22dest | 29.2 | 39,4 | 12 | * |
| D13_Clone04 | GmIRX14 (f5) | Δ1-156 and Δ436-502 | pET22dest | 36.3 | 21,0 | 7 | * |

Non-*Arabidopsis* CWGTs selected from the automated capillary electrophoresis results for SDS-PAGE, reported in the order of Fig. 3 for easy referral. Samples were selected on the basis of a yield above µg/mL, a purity above 27%, or by visual inspection of the results in ’gel mode’ (starred). Truncations and expression vectors are reported together with the yield and purity calculated by the Labchip GXII software. The well number is given in parenthesis after the protein name, to facilitate referral to Fig. 3, and a unique identifier refers to S4 Table.
