# Supplementary material for: Plant cell wall glycosyltransferases: High-throughput recombinant expression screening and general requirements for these challenging enzymes
Source: PLoS One. 2017 Jun 9;12(6):e0177591. doi: 10.1371/journal.pone.0177591 (PMC5466300; doi:10.1371/journal.pone.0177591)
Supplement: S2 Data — (PPTX) [file pone.0177591.s011.pptx]

## Slide 1
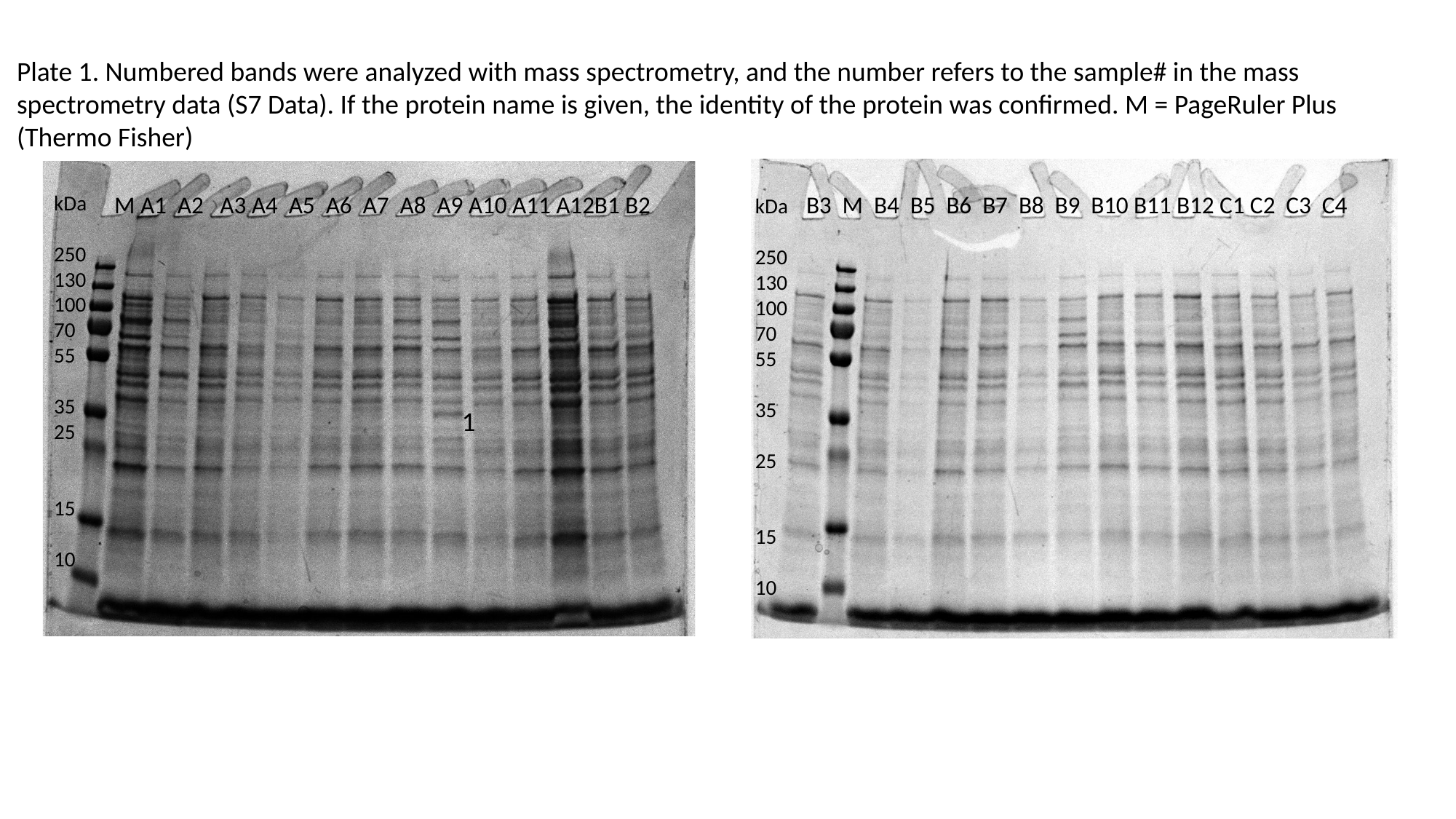

Plate 1. Numbered bands were analyzed with mass spectrometry, and the number refers to the sample# in the mass spectrometry data (S7 Data). If the protein name is given, the identity of the protein was confirmed. M = PageRuler Plus (Thermo Fisher)
kDa
250
130
100
70
55
35
25
15
10
M A1 A2 A3 A4 A5 A6 A7 A8 A9 A10 A11 A12B1 B2
B3 M B4 B5 B6 B7 B8 B9 B10 B11 B12 C1 C2 C3 C4
kDa
250
130
100
70
55
35
25
15
10
1

## Slide 2
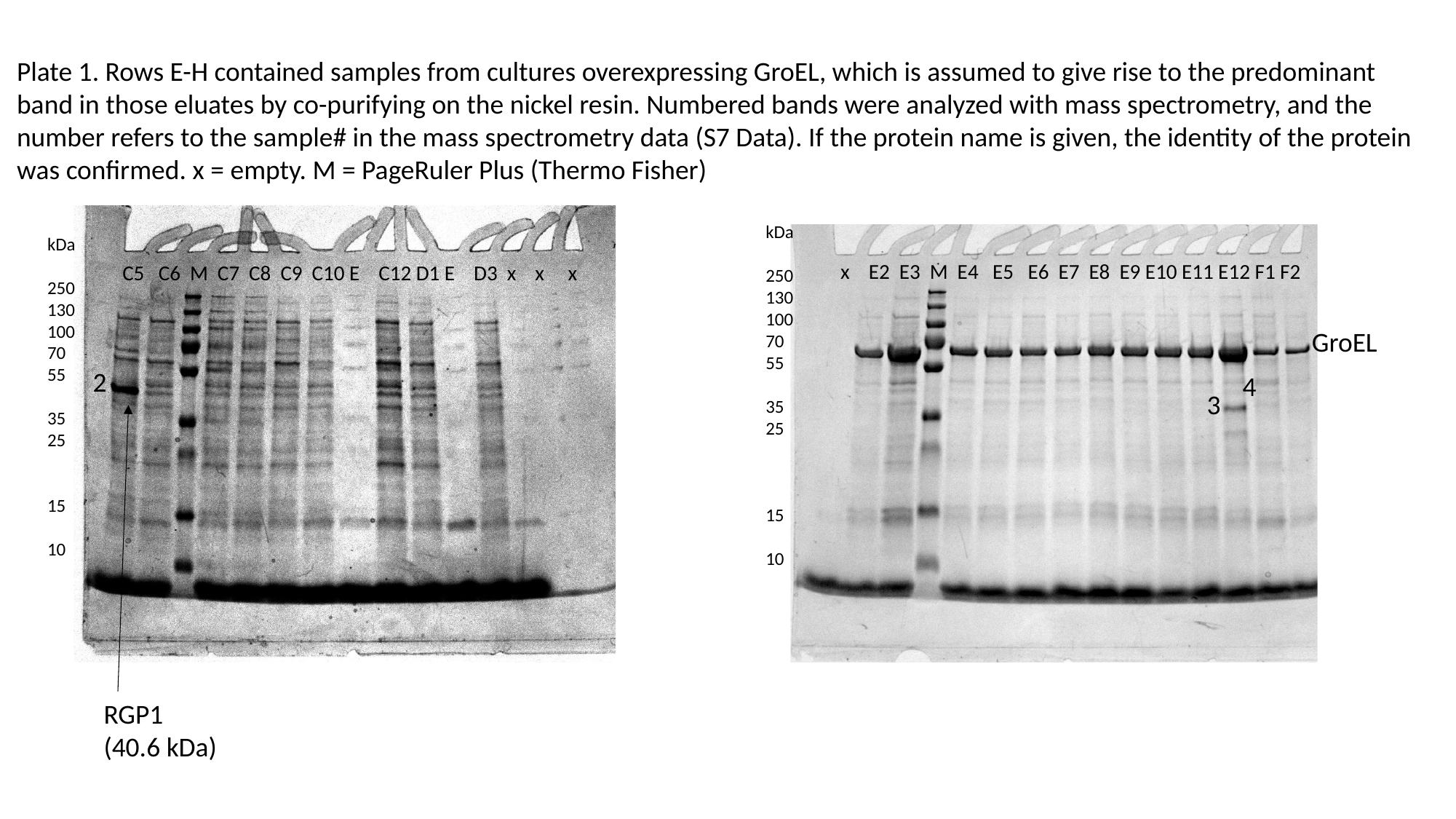

Plate 1. Rows E-H contained samples from cultures overexpressing GroEL, which is assumed to give rise to the predominant band in those eluates by co-purifying on the nickel resin. Numbered bands were analyzed with mass spectrometry, and the number refers to the sample# in the mass spectrometry data (S7 Data). If the protein name is given, the identity of the protein was confirmed. x = empty. M = PageRuler Plus (Thermo Fisher)
kDa
250
130
100
70
55
35
25
15
10
kDa
250
130
100
70
55
35
25
15
10
x E2 E3 M E4 E5 E6 E7 E8 E9 E10 E11 E12 F1 F2
C5 C6 M C7 C8 C9 C10 E C12 D1 E D3 x x x
GroEL
2
4
3
RGP1
(40.6 kDa)

## Slide 3
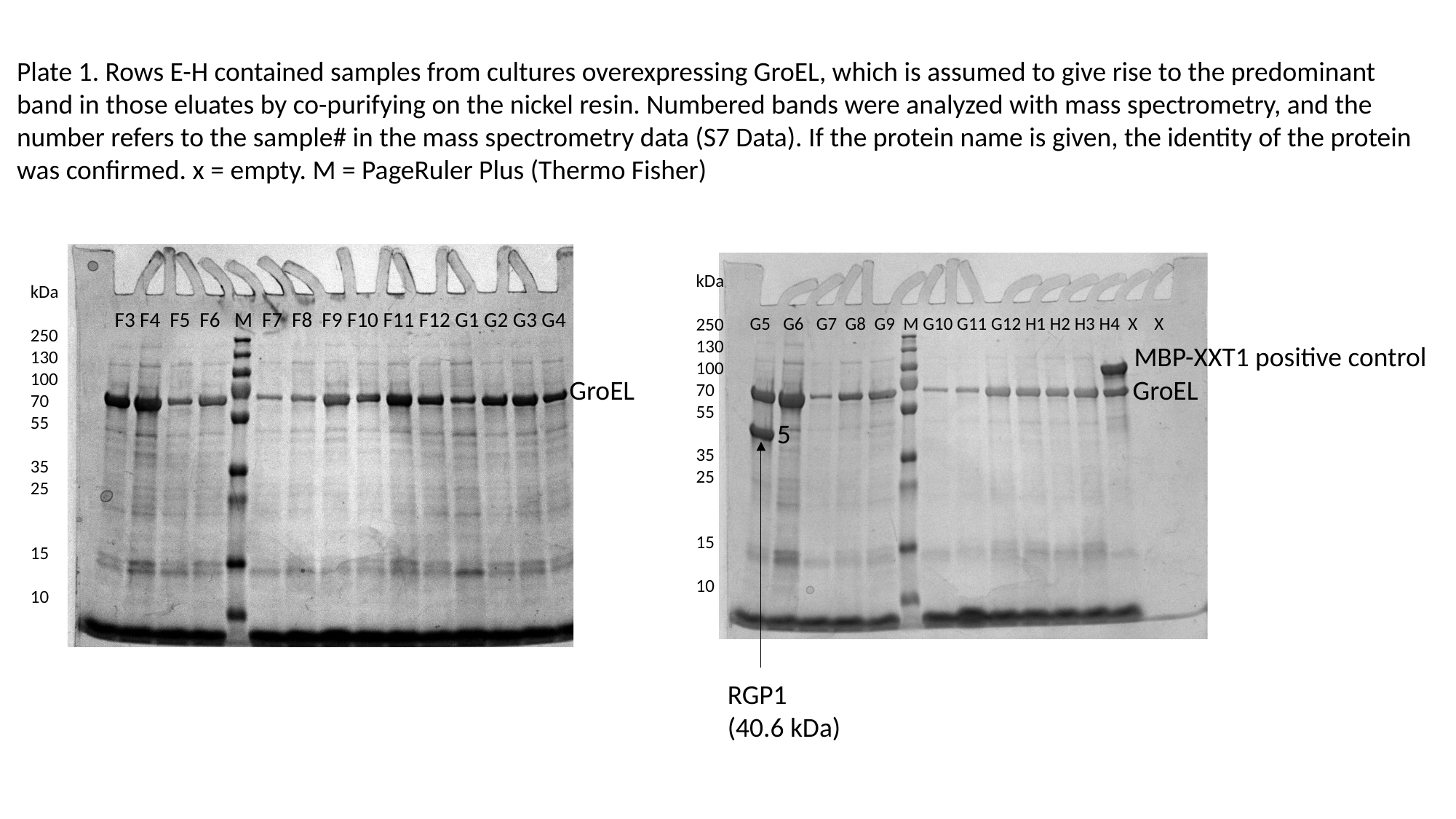

Plate 1. Rows E-H contained samples from cultures overexpressing GroEL, which is assumed to give rise to the predominant band in those eluates by co-purifying on the nickel resin. Numbered bands were analyzed with mass spectrometry, and the number refers to the sample# in the mass spectrometry data (S7 Data). If the protein name is given, the identity of the protein was confirmed. x = empty. M = PageRuler Plus (Thermo Fisher)
kDa
250
130
100
70
55
35
25
15
10
kDa
250
130
100
70
55
35
25
15
10
F3 F4 F5 F6 M F7 F8 F9 F10 F11 F12 G1 G2 G3 G4
G5 G6 G7 G8 G9 M G10 G11 G12 H1 H2 H3 H4 X X
MBP-XXT1 positive control
GroEL
GroEL
5
RGP1
(40.6 kDa)

## Slide 4
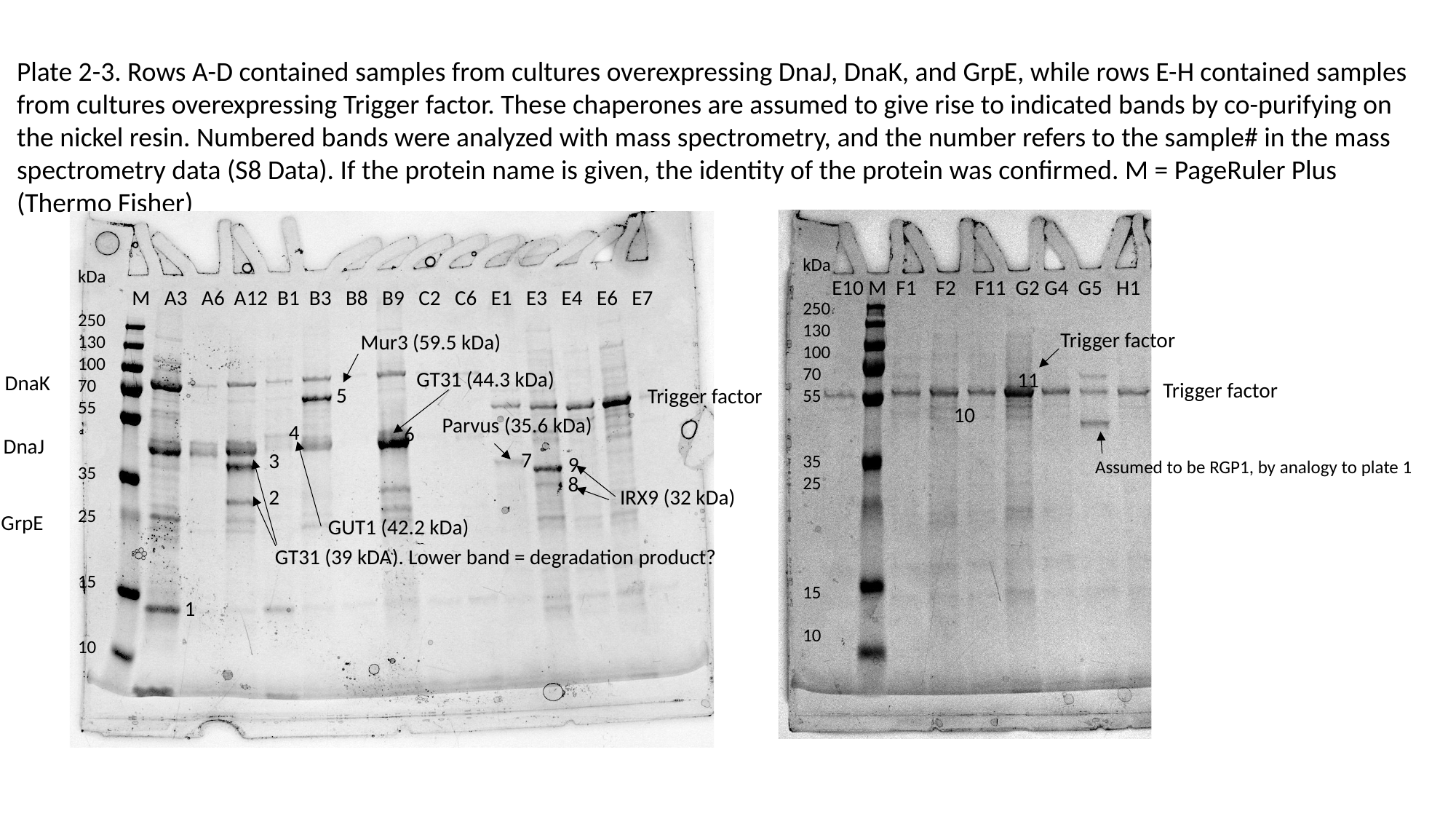

Plate 2-3. Rows A-D contained samples from cultures overexpressing DnaJ, DnaK, and GrpE, while rows E-H contained samples from cultures overexpressing Trigger factor. These chaperones are assumed to give rise to indicated bands by co-purifying on the nickel resin. Numbered bands were analyzed with mass spectrometry, and the number refers to the sample# in the mass spectrometry data (S8 Data). If the protein name is given, the identity of the protein was confirmed. M = PageRuler Plus (Thermo Fisher)
kDa
250
130
100
70
55
35
25
15
10
kDa
250
130
100
70
55
35
25
15
10
E10 M F1 F2 F11 G2 G4 G5 H1
M A3 A6 A12 B1 B3 B8 B9 C2 C6 E1 E3 E4 E6 E7
Trigger factor
Mur3 (59.5 kDa)
GT31 (44.3 kDa)
11
DnaK
Trigger factor
5
Trigger factor
10
Parvus (35.6 kDa)
4
6
DnaJ
7
 3
9
Assumed to be RGP1, by analogy to plate 1
8
IRX9 (32 kDa)
 2
GrpE
GUT1 (42.2 kDa)
GT31 (39 kDA). Lower band = degradation product?
1

## Slide 5
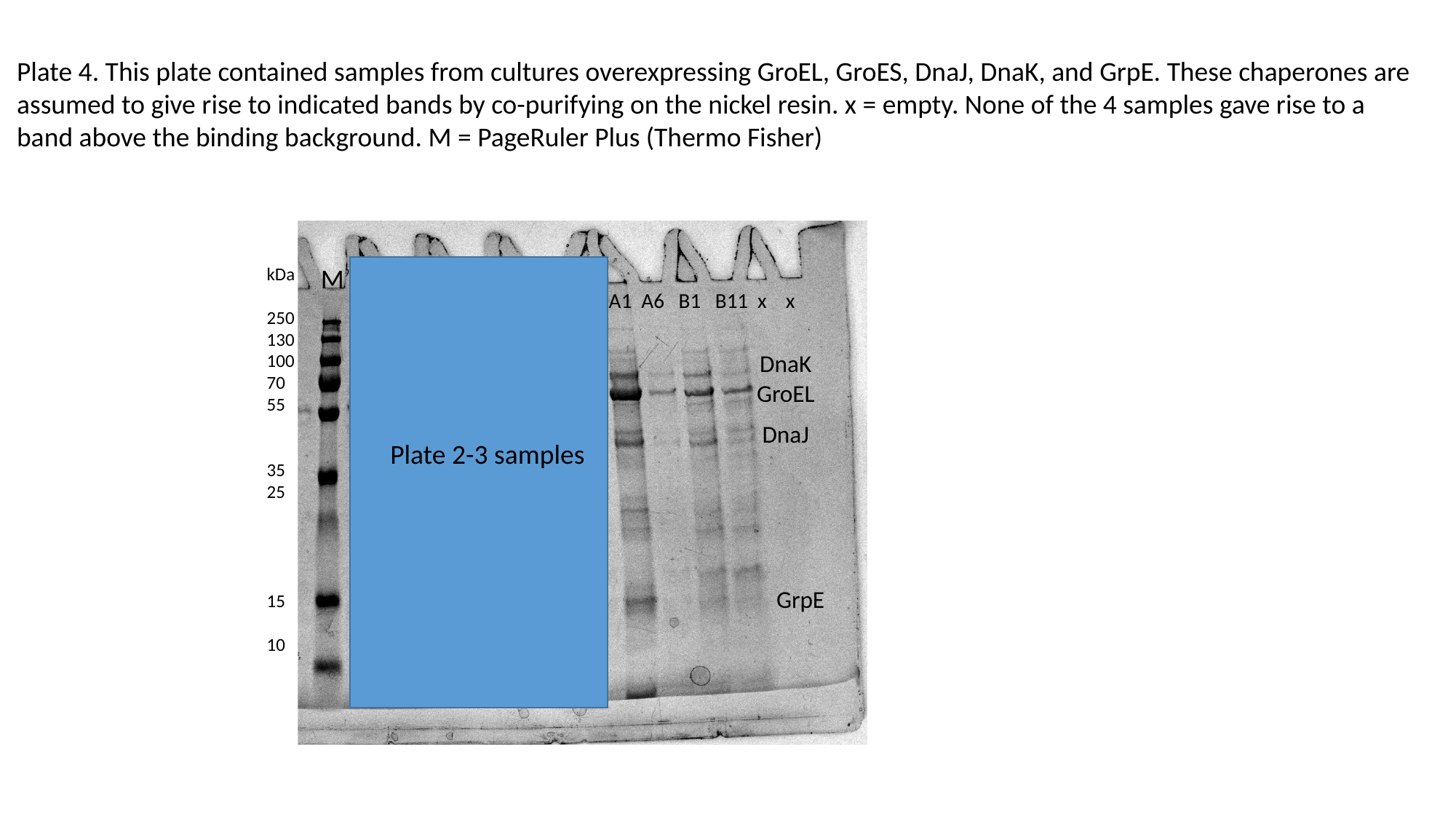

Plate 4. This plate contained samples from cultures overexpressing GroEL, GroES, DnaJ, DnaK, and GrpE. These chaperones are assumed to give rise to indicated bands by co-purifying on the nickel resin. x = empty. None of the 4 samples gave rise to a band above the binding background. M = PageRuler Plus (Thermo Fisher)
M
kDa
250
130
100
70
55
35
25
15
10
A1 A6 B1 B11 x x
DnaK
GroEL
DnaJ
Plate 2-3 samples
GrpE

## Slide 6
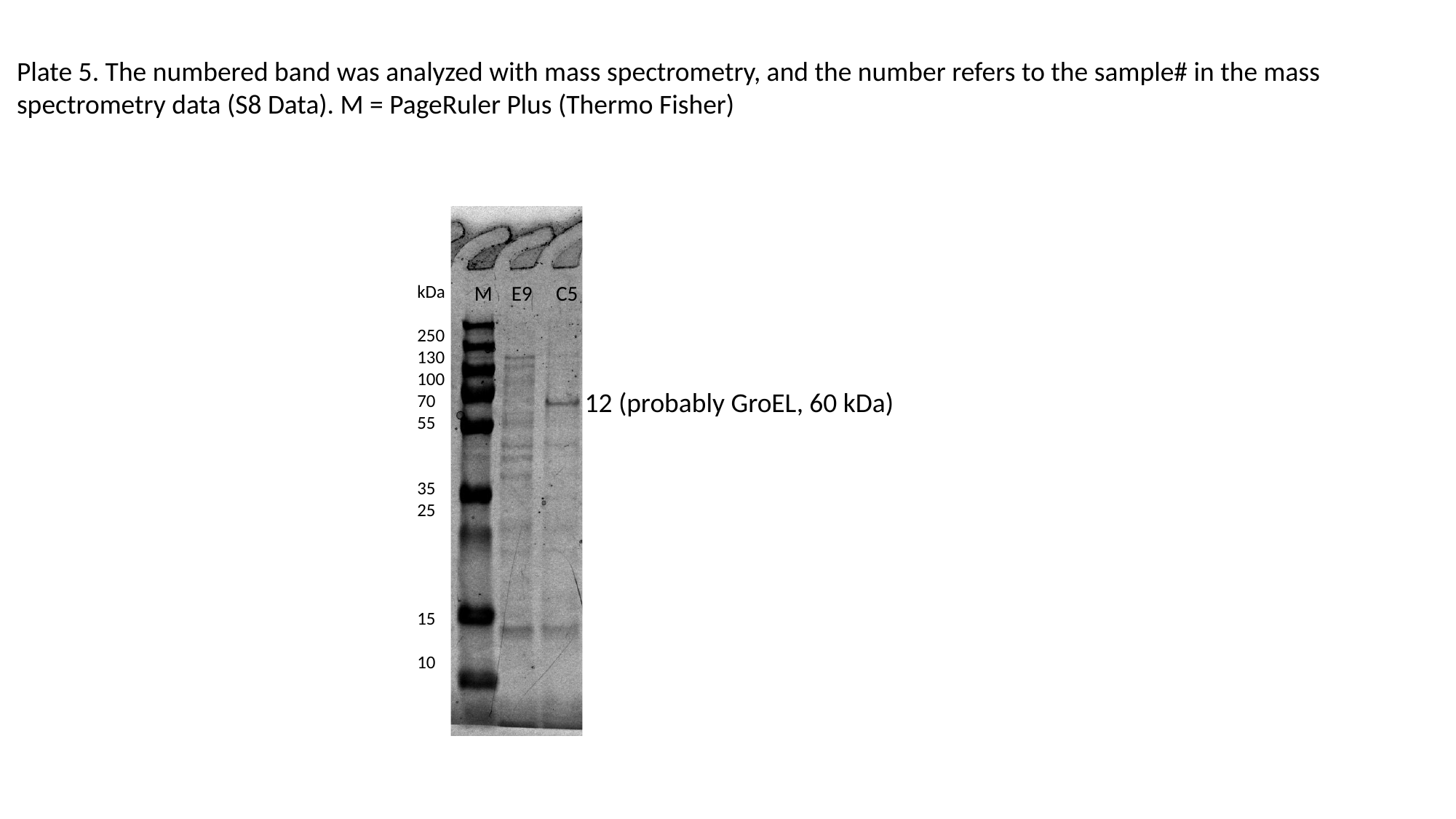

Plate 5. The numbered band was analyzed with mass spectrometry, and the number refers to the sample# in the mass spectrometry data (S8 Data). M = PageRuler Plus (Thermo Fisher)
kDa
250
130
100
70
55
35
25
15
10
M E9 C5
12 (probably GroEL, 60 kDa)

## Slide 7
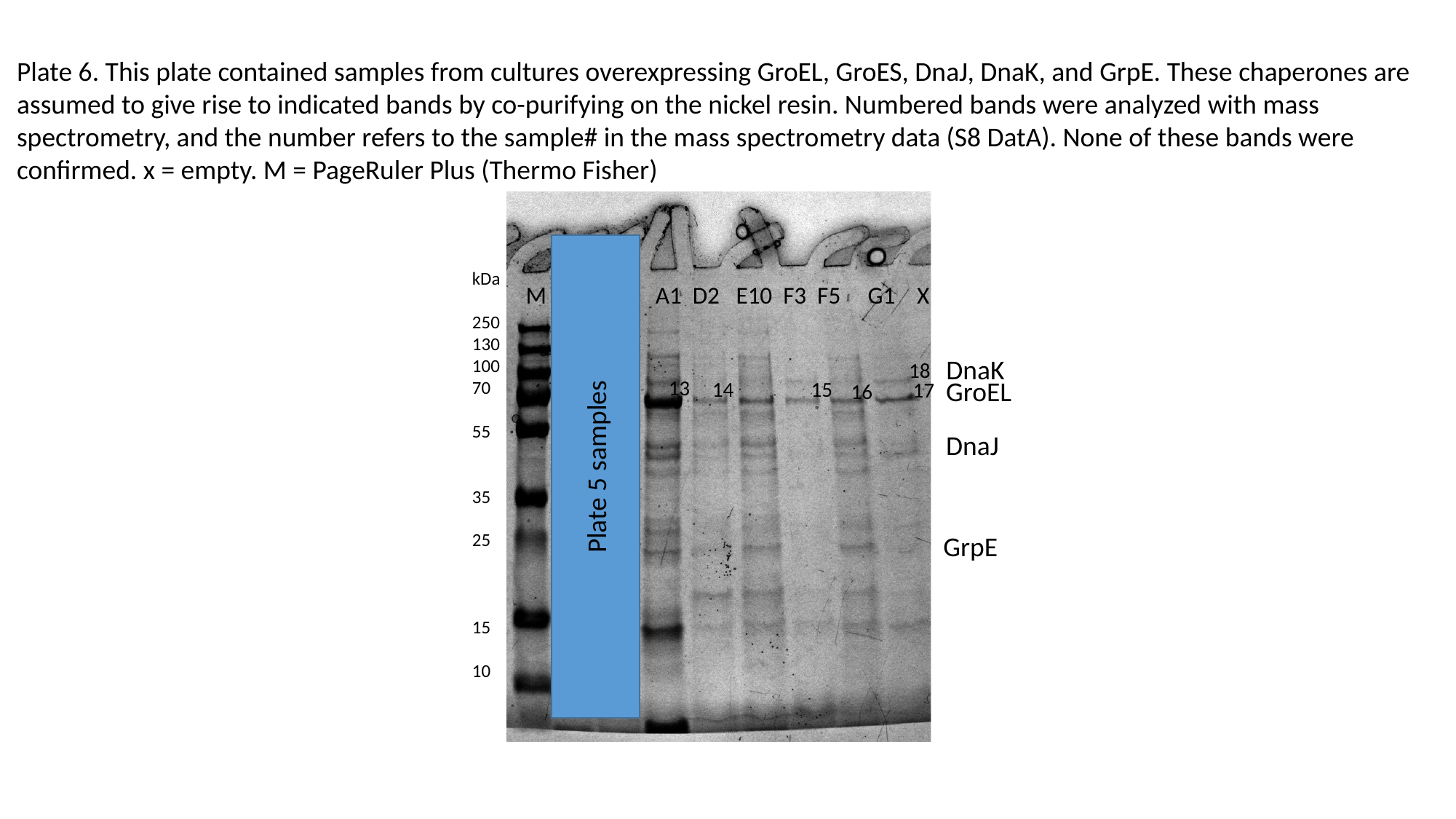

Plate 6. This plate contained samples from cultures overexpressing GroEL, GroES, DnaJ, DnaK, and GrpE. These chaperones are assumed to give rise to indicated bands by co-purifying on the nickel resin. Numbered bands were analyzed with mass spectrometry, and the number refers to the sample# in the mass spectrometry data (S8 DatA). None of these bands were confirmed. x = empty. M = PageRuler Plus (Thermo Fisher)
kDa
250
130
100
70
55
35
25
15
10
M A1 D2 E10 F3 F5 G1 X
DnaK
18
13
GroEL
15
14
17
16
DnaJ
Plate 5 samples
GrpE

## Slide 8
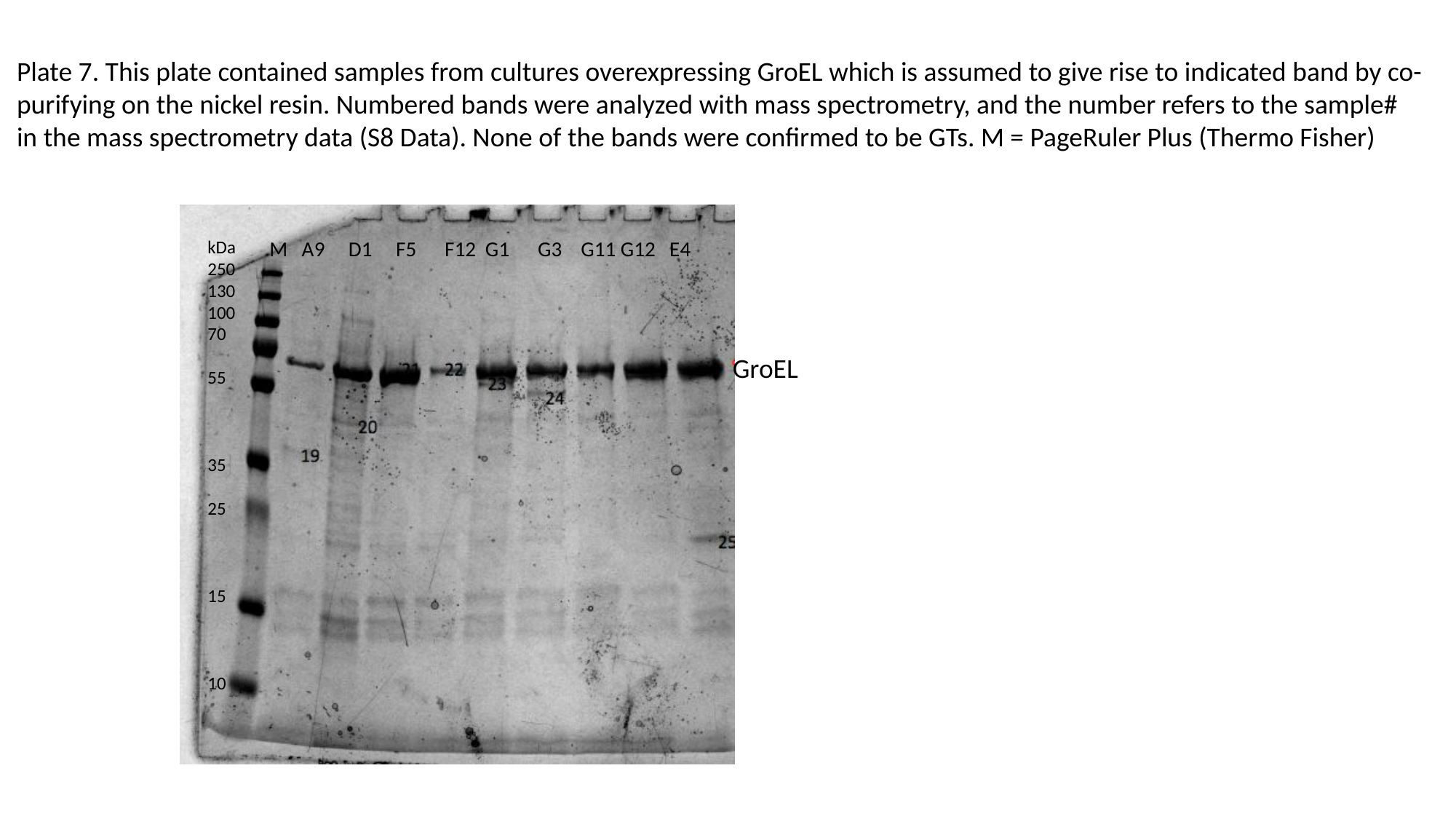

Plate 7. This plate contained samples from cultures overexpressing GroEL which is assumed to give rise to indicated band by co-purifying on the nickel resin. Numbered bands were analyzed with mass spectrometry, and the number refers to the sample# in the mass spectrometry data (S8 Data). None of the bands were confirmed to be GTs. M = PageRuler Plus (Thermo Fisher)
kDa
250
130
100
70
55
35
25
15
10
M A9 D1 F5 F12 G1 G3 G11 G12 E4
GroEL

## Slide 9
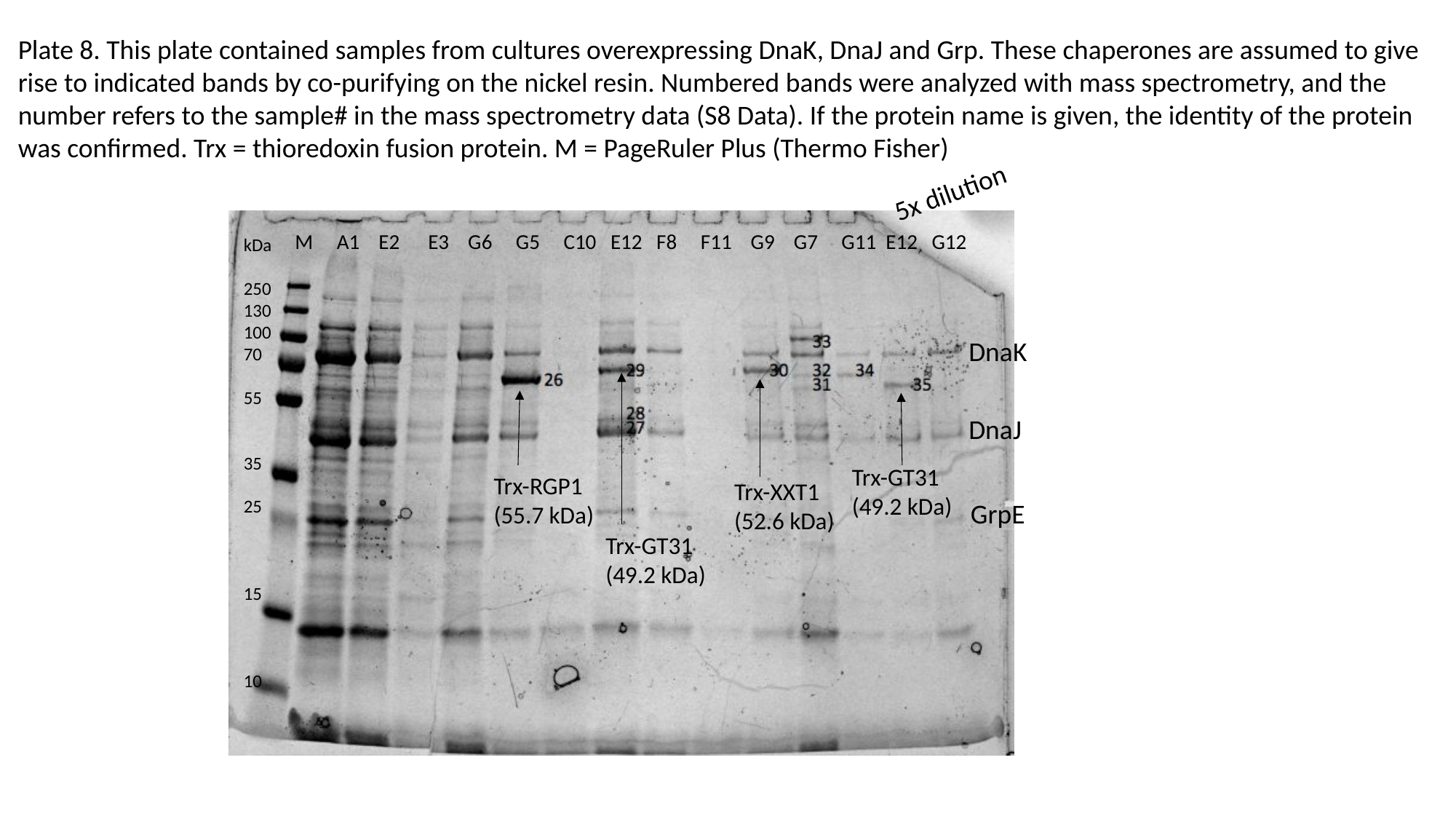

Plate 8. This plate contained samples from cultures overexpressing DnaK, DnaJ and Grp. These chaperones are assumed to give rise to indicated bands by co-purifying on the nickel resin. Numbered bands were analyzed with mass spectrometry, and the number refers to the sample# in the mass spectrometry data (S8 Data). If the protein name is given, the identity of the protein was confirmed. Trx = thioredoxin fusion protein. M = PageRuler Plus (Thermo Fisher)
5x dilution
M A1 E2 E3 G6 G5 C10 E12 F8 F11 G9 G7 G11 E12 G12
kDa
250
130
100
70
55
35
25
15
10
DnaK
DnaJ
Trx-GT31
(49.2 kDa)
Trx-RGP1
(55.7 kDa)
Trx-XXT1
(52.6 kDa)
GrpE
Trx-GT31
(49.2 kDa)

## Slide 10
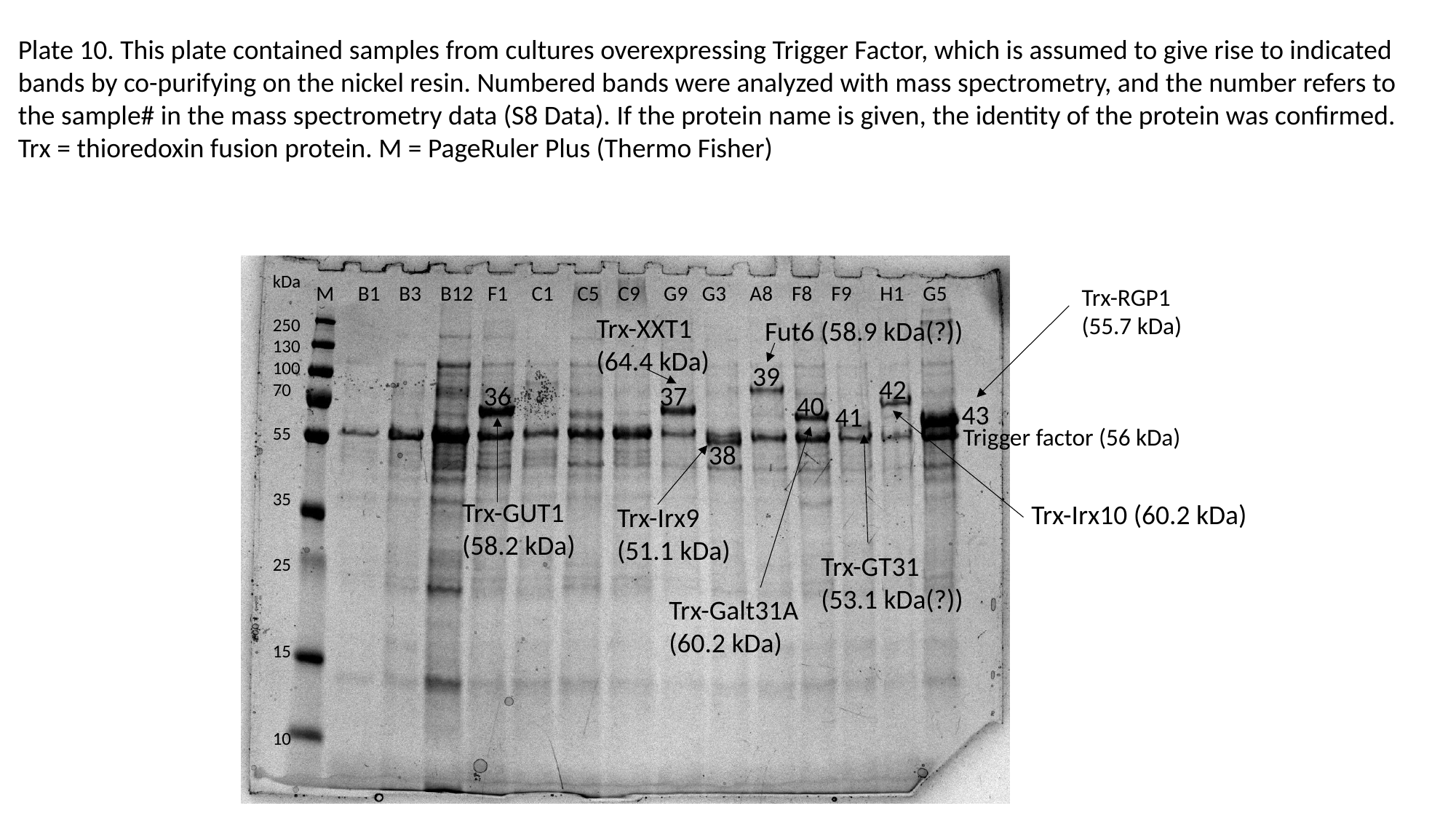

Plate 10. This plate contained samples from cultures overexpressing Trigger Factor, which is assumed to give rise to indicated bands by co-purifying on the nickel resin. Numbered bands were analyzed with mass spectrometry, and the number refers to the sample# in the mass spectrometry data (S8 Data). If the protein name is given, the identity of the protein was confirmed. Trx = thioredoxin fusion protein. M = PageRuler Plus (Thermo Fisher)
kDa
250
130
100
70
55
35
25
15
10
M B1 B3 B12 F1 C1 C5 C9 G9 G3 A8 F8 F9 H1 G5
Trx-RGP1
(55.7 kDa)
Trx-XXT1
(64.4 kDa)
Fut6 (58.9 kDa(?))
39
42
36
37
40
43
41
Trigger factor (56 kDa)
38
Trx-GUT1 (58.2 kDa)
Trx-Irx10 (60.2 kDa)
Trx-Irx9
(51.1 kDa)
Trx-GT31
(53.1 kDa(?))
Trx-Galt31A
(60.2 kDa)
