# Supplementary material for: Plant cell wall glycosyltransferases: High-throughput recombinant expression screening and general requirements for these challenging enzymes
Source: PLoS One. 2017 Jun 9;12(6):e0177591. doi: 10.1371/journal.pone.0177591 (PMC5466300; doi:10.1371/journal.pone.0177591)
Supplement: S1 List — (DOCX) [file pone.0177591.s016.docx]

**S1 List. The CWGT-customized lysis buffer screen.**

Standard lysis buffer: 50 mM HEPES pH 8.0, 250 mM NaCl and 5 mM MgSO_4_

1. 100 mM Tris, 10% glycerol, 5mM MgSO_4_, 5mM UDP, pH 7.6
2. 100 mM Tris, 50 mM LiCl, 5mM MgSO_4_, pH 7.6
3. 100 mM HEPES, 50 mM (NH4)_2_SO_4_, 10% glycerol, 5mM MgSO_4_, pH 7.0
4. 100 mM HEPES, 100 mM KCl, 5mM MgSO_4_, pH 7.0
5. 100 mM Tris, 50 mM NaCl, 10% isopropanol, 5mM MgSO_4_, pH 8.2
6. 100 mM K_2_HPO_4_/K_2_HPO_4_, 50 mM (NH4)_2_SO_4_, 1% Triton X-100, 5mM MgSO_4_, pH 6.0
7. 100 mM triethanolamine, 100 mM KCl, 10 mM DTT, 5mM MgSO_4_, pH 8.5
8. 100 mM Tris, 80 mM sodium glutamate, 10 mM DTT, 5mM MgSO_4_, pH 8.2
9. 250 mM KH_2_PO_4_/K_2_HPO_4_, 0.1% CHAPS, 5mM MgSO_4_, pH 6.0
10. 100 mM triethanolamine, 50 mM LiCl, 5 mM EDTA, pH 8.5
11. 100 mM sodium acetate, 100 mM glutamine, 10 mM DTT, 5mM MgSO_4_ pH 5.5
12. 100 mM sodium acetate, 100 mM KCl, 0.1% n-octyl-β-D-glucoside, 5mM MgSO_4_ pH 5.5
13. 100 mM HEPES, 1 M MgSO4, pH 7.0
14. 100 mM HEPES, 50 mM LiCl, 0.1% CHAPS, 5mM MgSO_4_, pH 7.0
15. 100 mM KH_2_PO_4_/K_2_HPO_4_, 2.5 mM ZnCl2, 5mM MgSO_4_, pH 4.3
16. 100 mM Tris, 50 mM NaCl, 5 mM calcium acetate, 5mM MgSO_4_, pH 7.6
17. 100 mM triethanolamine, 50 mM (NH4)_2_SO_4_, 10 mM MgSO4, 5mM UDP, pH 8.5
18. 100 mM Tris, 100 mM KCl, 2 mM EDTA, 1% Triton X-100, pH 8.2
19. 100 mM sodium acetate, 1M MgSO4, pH 5.5
20. 100 mM Tris, 2M NaCl, 0.1% n-octyl-β-D-glucoside, 5mM MgSO_4_, pH 7.6
21. 100 mM Tris, 1 M (NH4)_2_SO_4_, 10 mM DTT, 5mM MgSO_4_, pH 8.2
22. 100 mM sodium acetate, 50 mM LiCl, 5 mM calcium acetate, 5mM MgSO_4_, 5 mM UDP, pH 5.5
23. 100 mM HEPES, 80 mM sodium glutamate, 5 mM DTT, 5mM MgSO_4_, pH 7.0
24. 100 mM triethanolamine, 80 mM sodium glutamate, 0.02% n-octyl-β-D-glucoside, 10% glycerol, 5mM MgSO_4_, pH 8.5
25. 100 mM Tris, 50 mM NaCl, 100 mM urea, 5mM MgSO_4_, 5mM UDP, pH 8.2
26. 100 mM triethanolamine, 100 mM KCl, 0.05% dextran sulfate, pH 8.5
27. 100 mM KH2PO4/K2HPO4, 50 mM (NH4)_2_SO_4_, 0.05% dextran sulfate, pH 6.0
28. 100 mM HEPES, 50 mM LiCl, 0.1% deoxycholate, pH 7.0
29. 100 mM Tris, 100 mM KCl, 0.1% deoxycholate, 25% glycerol, pH 7.6
30. 100 mM potassium acetate, 50 mM NaCl, 0.05% dextran sulfate, 0.1% CHAPS, pH 5.5
